# Effect of an enteral amino acid blend on muscle and gut functionality in critically ill patients: a proof-of-concept randomized controlled trial

**DOI:** 10.1186/s13054-022-04232-5

**Published:** 2022-11-17

**Authors:** Nicholas Heming, Robert Carlier, Helene Prigent, Ahmed Mekki, Camille Jousset, Frederic Lofaso, Xavier Ambrosi, Rania Bounab, Virginie Maxime, Arnaud Mansart, Pascal Crenn, Pierre Moine, Fabien Foltzer, Bernard Cuenoud, Tobias Konz, John Corthesy, Maurice Beaumont, Mickaël Hartweg, Claudia Roessle, Jean-Charles Preiser, Denis Breuillé, Djillali Annane

**Affiliations:** 1 General Intensive Care Unit, Raymond Poincaré Hospital (AP-HP), University of Versailles Saint-Quentin en Yvelines, University Paris Saclay, 104, Boulevard Raymond Poincaré, 92380 Garches, France; 2 Laboratory of Infection and Inflammation - U1173, School of Medicine Simone Veil, INSERM, University Versailles Saint Quentin - University Paris Saclay, Garches, France; 3 FHU SEPSIS (Saclay and Paris Seine Nord Endeavour to PerSonalize Interventions for Sepsis), 92380 Garches, France; 4 RHU RECORDS (Rapid rEcognition of CORticosteroiD Resistant or Sensitive Sepsis), 92380 Garches, France; 5 Department of Radiology, APHP, DMU Smart Imaging, GH Université Paris-Saclay, Hôpital Raymond Poincaré, Garches, France; 6 Department of Physiology-AP-HP, Hôpital Raymond-Poincaré, Garches, France; 7 UFR des Sciences de la Santé Simone-Veil, Université de Versailles Saint-Quentin-en-Yvelines, Montigny-le-Bretonneux, France; 8 Department of Anesthesiology and Intensive Care Medicine, University Hospital of Nantes, Nantes, France; 9 Clinical Nutrition Unit and FHU Hepatinov, Hôpital Raymond Poincaré, APHP Université Paris Saclay, Garches, France; 10 Nestlé Research, Société de Produits de Nestlé, Lausanne, Switzerland; 11 Translation Research, Nestlé Health Science, Lausanne, Switzerland; 12 Nutrition Team, Erasme University Hospital, Université Libre de Bruxelles, 1070 Brussels, Belgium

**Keywords:** Sepsis, Critical illness, Protein balance, Intensive care unit, Nutrition

## Abstract

**Background:**

A defining feature of prolonged critical illness is muscle wasting, leading to impaired recovery. Supplementation with a tailored blend of amino acids may bolster the innate gut defence, promote intestinal mucosa repair and limit muscle loss.

**Methods:**

This was a monocentric, randomized, double-blind, placebo-controlled study that included patients with sepsis or acute respiratory distress syndrome. Patients received a specific combination of five amino acids or placebo mixed with enteral feeding for 21 days. Markers of renal function, gut barrier structure and functionality were collected at baseline and 1, 2, 3 and 8 weeks after randomization. Muscle structure and function were assessed through MRI measurements of the anterior quadriceps volume and by twitch airway pressure. Data were compared between groups relative to the baseline.

**Results:**

Thirty-five critically ill patients were randomized. The amino acid blend did not impair urine output, blood creatinine levels or creatinine clearance. Plasma citrulline levels increased significantly along the treatment period in the amino acid group (difference in means [95% CI] 5.86 [1.72; 10.00] nmol/mL *P* = 0.007). Alanine aminotransferase and alkaline phosphatase concentrations were lower in the amino acid group than in the placebo group at one week (ratio of means 0.5 [0.29; 0.86] (*P* = 0.015) and 0.73 [0.57; 0.94] (*P* = 0.015), respectively). Twitch airway pressure and volume of the anterior quadriceps were greater in the amino acid group than in the placebo group 3 weeks after randomization (difference in means 10.6 [0.99; 20.20] cmH_2_0 (*P* = 0.035) and 3.12 [0.5; 5.73] cm^3^/kg (*P* = 0.022), respectively).

**Conclusions:**

Amino acid supplementation increased plasma citrulline levels, reduced alanine aminotransferase and alkaline phosphatase levels, and improved twitch airway pressure and anterior quadriceps volume.

*Trial registration* ClinicalTrials.gov, NCT02968836. Registered November 21, 2016.

**Supplementary Information:**

The online version contains supplementary material available at 10.1186/s13054-022-04232-5.

## Introduction

A small proportion of critically ill patients require prolonged intensive care unit (ICU) stays; however, these patients account for a disproportionate use of critical care resources [1]. This population is at increased risk of muscle mass and muscle strength loss and subsequent impaired recovery [2–4]. Systemic inflammation, such as that occurring in sepsis or acute respiratory distress syndrome (ARDS), leads to increased protein synthesis in the splanchnic area, especially in the gut and liver [5, 6]. The liver produces acute phase proteins, key components of the innate immune response, in response to inflammation [7]. In parallel, the ensuing oxidative stress induces a strong increase in glutathione synthesis in all tissues, especially the liver [8]. During sepsis, the consumption of cysteine increases twofold [8]. Amino acids are also implicated in the repair of the intestinal mucosa and in the provision of energy to bolster the innate defence of the gut [9, 10]. Stress therefore leads to increased demand for amino acids by the gut and the liver. Muscles may act as protein reservoirs in the acute setting, delivering amino acids to the splanchnic area through the activation of muscle protein catabolism [11]. Reducing muscle loss and promoting lean body mass recovery in critically ill patients is of utmost importance. Due to a lack of adequately powered clinical trials, the benefit–risk balance of administrating higher amounts of proteins or amino acids during critical illness is unknown [12]. Increasing energy and protein intake has little effect on limiting muscle wasting or improving quality of life [13–15]. In contrast, excessive caloric intake may lead to negative effects on recovery and ICU-acquired muscle weakness, possibly by stimulating the autophagy of myofibres [16–18]. Additional trials exploring the optimal dose of proteins/amino acids are therefore warranted.

Preclinical studies in sepsis have shown that specific amino acid supplementation decreases muscle protein breakdown and increases muscle protein synthesis [7]. Serine, threonine and cysteine promote acute phase protein biosynthesis in the splanchnic area [7]. Threonine stimulates the production of intestinal mucins, promoting gut barrier function [19, 20]. Mucins contain a high proportion of threonine, cysteine, proline and serine, all implicated in the maintenance of the gut barrier function [21]. Cysteine affects the antioxidant response through its role in glutathione synthesis. Leucine may have a direct positive impact on muscle protein synthesis [22]. However, data regarding the effect of amino acid supplementation in critically ill patients are sparse.

The aim of this proof-of-concept study was to demonstrate whether enteral supplementation by a specifically tailored blend of threonine, cysteine, proline, serine and leucine would be safe and exert a positive action on muscle mass, strength and function as well as on gut function in ICU patients.

## Methods

### Study design

This proof-of-concept, randomized, double-blind, placebo-controlled trial was performed on two parallel groups. The trial was conducted at the medical ICU at the Raymond Poincaré University Hospital (Assistance Publique Hôpitaux de Paris, Garches, France). The trial protocol was approved by an independent ethics committee (Comité de Protection des Personnes d’Ile de France XI, Saint-Germain-en-Laye, France), was registered with ClinicalTrials.gov, number NCT02968836 (registered November 21, 2016) and conducted in accordance with French law and the Declaration of Helsinki.

### Participants

Patients were eligible for inclusion within 72 h of ICU admission if they were aged 18 and over, met criteria for sepsis [23] or, following an amendment to the protocol in December 2017, ARDS [24], and had an expected length of stay in the ICU or in the intermediate care unit of at least 21 days. Patients were excluded if they exhibited muscle mass loss due to previous hospitalization or were cachectic (body mass index < 18.5 kg/m^2^ or unintentional weight loss either > 10% of habitual weight indefinite of time, or > 5% over 3 months) [25, 26], intolerant to enteral feeding (vomiting or diarrhoea) [27], receiving long-term parenteral feeding, had a history of chronic renal failure (chronic kidney disease (CKD) stages 4 or 5), chronic liver disease (cirrhosis, liver transplant, liver cancer, etc.), or chronic intestinal disease (ulcerative colitis, Crohn's disease, short bowel syndrome, etc.), had been treated by radiotherapy or chemotherapy for cancer, had a pacemaker or metal implants contraindicating magnetic resonance imaging (MRI), or were pregnant. Patients receiving neuromuscular-blocking agents were also excluded as well as patients without social security, patients under guardianship, patients expected not to comply with study procedures, and patients having participated in another clinical trial within the last 4 weeks.

Written informed consent was obtained from each patient or from his or her legally authorized representative if the patient was unable to provide consent. Alternatively, deferred written informed consent was obtained from the patients.

### Randomization and masking

Eligible patients with sepsis or ARDS were randomly assigned in a 1:1 ratio to receive a blend of 5 amino acids (threonine, cysteine, proline, serine and leucine) or a placebo. The group receiving the amino acid blend will be called the “amino acid group” throughout the manuscript. The randomization list was generated by a computer, and randomization was stratified by age (≤ 50 years or > 50 years) and balanced using the dynamic allocation method (Medidata RTSM, second-best probability set to 15%). The investigational product and the placebo were similar at all points, were produced in powder form and were enclosed in identically looking sticks.

Participants, ICU staff, investigators, pharmacists, statisticians and sponsors remained blinded to the nature of the product (investigational or placebo) being administered throughout the study period.

### Procedures

#### Study intervention

Amino acids were administered through the enteral route as a supplement to enteral nutrition. A single dose of active treatment contained 3 g of threonine, 1.3 g of proline, 2.5 g of serine, 2 g of cysteine, 2.3 g of leucine, and 10.5 g of maltodextrin. Each dose of the investigational product was mixed in a bottle containing 500 ml of Isosource Energy® (Isosource Energy®-Nestle Health Science, France), accounting for an additional 88 kcal. Isosource Energy® contains 61.0 g of protein per litre and 1500 kcal per litre. The investigational product was continuously administered over a 21-day period or until enteral nutrition was interrupted by the physician in charge of the patient. The matching isocaloric placebo containing 22 g maltodextrin was administered following the exact same method as the investigational product.

Patient management adhered to international guidelines [28]. Albumin was administered to septic patients previously treated with large quantities of crystalloids [29]. Enteral nutrition was initiated as soon as feasible (within 24 h of intubation). Enteral nutrition was administered through a nasogastric feeding tube. Patients were placed in a semirecumbent position, unless medically contraindicated. The tube position was mandatorily checked before the initiation of enteral feeding. Nutritional targets ranged from 25 to 30 kcal/kg/day [30, 31]. Enteral nutrition was administered continuously. Nutrition was set at the required flow rate to reach the target calorie intake on day 1. Vomiting or regurgitation was managed by transiently reducing the feed flow rate or administrating prokinetic drugs. Water, trace elements, vitamins and minerals were administered as deemed necessary by the attending physician. The quantities of calories and proteins ingested after oral nutrition resumed were not measured.

Patients in our institution benefit from standardized early rehabilitation, beginning with passive motion exercises, passive or active exercises followed whenever possible by fully active muscle exercises, including in-bed-cycling, transfer to a chair, standing and ultimately walking [32].

#### Screening and follow-up visit (Additional file 1: Fig. S1)

A screening visit was performed at ICU admission to determine eligibility for the study. A baseline visit (V1) was performed within 72 h of admission. Follow-up visits (V2 to V7) were conducted 7, 14, 21, 60, 180 and 365 days after randomization. We recorded urine output, serum creatinine and glomerular filtration rate during the baseline visit and up to 21 days after randomization [33]. Blood chemistry was assessed at every visit until day 60. We measured quadriceps extension isometric strength and twitch airway pressure in response to magnetic stimulation as well as forced vital capacity and maximal inspiratory and expiratory pressures in nonsedated patients at every visit. Anterior quadriceps volume was measured at every visit using a 3-Tesla MR imaging system (MR750; GE Healthcare, Milwaukee, WI, USA). Anterior quadriceps volume was measured semi-automatically using isometric T1 weighted 3D images. Data are presented as the mean value of both legs. Following analysis of the overall results, we corrected for peripheral (muscle) oedema by indexing the quadriceps muscle volume to the patient’s weight, measured on the same day as the MRI was performed.

Quadriceps force was measured using magnetic stimulation of the femoral nerve [34]. Briefly, patients were studied while in the supine position with the knee flexed at 90° [35]. The ankle was attached to the arm of a dynamometer by means of an inextensible ankle trap. Stimulation was performed using a 45-mm figure-of-eight coil (Magstim 200, The Magstim Limited Company, Whitland, UK) [36]. The coil was oriented tangentially to the surface of the femoral triangle, lateral to the femoral artery. To optimize the position of the coil, several stimulations at 70% of maximal output were performed until optimal quadriceps response was determined. Four stimuli at 100% stimulator output were subsequently performed, and the maximal value of muscle extension was recorded. The procedure was repeated on both limbs.

Diaphragm muscle strength was assessed by measuring twitch airway pressure in response to magnetic stimulation of the phrenic nerve [37, 38]. Briefly, patients were placed in a reclining position and breathed through a flanged mouthpiece or directly through the endotracheal tube and a three-way nonrebreathing valve. The mouthpiece or the endotracheal tube was connected to a number 2 Fleisch pneumotachograph (Fleisch, Lausanne, Switzerland) and a differential pressure transducer (MP45 ± 100 cmH2O; Validyne Engineering Corp., Northridge, CA, USA). Twitch mouth pressure was measured after cervical magnetic stimulation via a 90-mm circular coil powered by a Magstim stimulator. To optimize the coil position, several stimulations at 70% of maximal output were performed over the spinal processes between C5 and C7 until an optimal diaphragmatic response was determined. To avoid upper airway collapse and/or glottis closure, the phrenic nerve was stimulated once a predetermined − 5 cmH_2_O inspiratory pressure trigger was reached [38, 39]. In the absence of spontaneous breathing, the tracheal tube was momentarily occluded at end-expiration before phrenic nerve stimulation [37]. A total of 4 stimulations were performed at maximal stimulator output, and the maximal values were recorded for analysis. A 30 s delay was programmed between stimulations to prevent potentiation [40].

Forced vital capacity was measured through a flanged mouthpiece or the endotracheal tube using a portable spirometer (SpirobankII, MIR France, Langlade, France) [41]. For each parameter, 3 measures were performed, and the best value was recorded.

Muscle protein catabolism was assessed by measuring urine 3-methyl histidine up until day 60 [42].

Gut barrier structure and functionality were assessed up to 60 days after randomization. Enterocyte injury was assessed by measuring plasma I-FABP (intestinal fatty acid-binding protein) [43]. Functional enterocyte mass was assessed by measuring plasma citrulline concentration [44]. Inflammatory status was assessed up to 60 days after randomization by the following measurements: calprotectin in faeces and plasma levels of C-reactive protein (CRP), procalcitonin, fibrinogen, ferritin and prealbumin. Liver function was assessed by measuring albumin, alanine aminotransferase, alkaline phosphatase and total bilirubin in plasma using an Architect Ci4100 analyser from Abbot Laboratories (Chicago, Illinois, USA) composed of a clinical chemistry module C4000 and an immunoassay module i1000SR. Each analyte was prepared following the manufacturer’s specific kit, references were 7D62 for cholesterol, 6L45 for total bilirubin, 8L92 for alanine aminotransferase, 7D55 for alkaline phosphatase and 7K59 for ferritin. Plasma amino acid concentrations were measured by ion exchange chromatography with spectrophotometric detection after ninhydrin derivatization [45]. Extensive nutrient profiling was performed until day 60 after randomization. The nutrient profiling consisted of quantitatively measuring fatty acids (in plasma as well as in red blood cells), water-soluble vitamins, fat-soluble vitamins, organic acids such as 2-ketobuyric acid, 3-methyl-2-oxobutyric acid, 3-methyl-2-oxopentanoic acid and 4-methyl-2-oxopentanoic acid, ascorbic acid and minerals with different analytical methods. In brief, a mineral analysis was performed with 150 µL of plasma diluted 1:10 in a diluent solution containing 5% 1-butanol, 0.05% EDTA, 0.05% Triton X-100, and 1% ammonium hydroxide. This analysis was performed using an 8800 ICP‒MS/MS spectrometer (Agilent Technologies, Tokyo, Japan) operated in low matrix plasma mode.

### Outcomes

Since this was a proof-of-concept exploratory trial, we did not assess any of the predefined outcomes hierarchically.

At every visit, efficacy was assessed with respect to muscle functionality (diaphragm and quadriceps strength and mass, and muscle protein catabolism), gut barrier structure and functionality, inflammatory status, liver biochemical parameters, nutrient profile and general recovery in terms of ICU-free survival, ventilation-free days and time to recover to walking without aid.

The safety evaluation focused primarily on renal function, which was assessed by measuring urine output, serum creatinine levels and creatinine clearance at each study visit.

### Statistical analysis

As this was an exploratory study, no formal sample size calculation was undertaken; by the rule of thumb, we determined a priori that recruitment proceed until a sample of 30 patients [46, 47] had completed follow-up at day 21. All endpoints were analysed separately without adjusting for multiplicity. Analysis was by intention to treat.

Mean changes from baseline at days 7, 14, 21 and 60 were analysed using a restricted maximum likelihood (REML)-based repeated measures approach. Baseline, treatment group, visit, age and sex were considered fixed effects, while within-subject variability was controlled for by considering the subject as a random effect. An unstructured covariance structure was used to model the within-patient errors; the covariance structure converging to the best fit, as determined by Akaike’s information criterion, was used in the full model. The Kenward–Roger approximation was used to estimate denominator degrees of freedom. The normality and homoscedasticity of variables were checked using the Shapiro‒Wilk normality test. The length of stay in the ICU was compared using the log-rank test.

All analyses were conducted with SAS statistical software, version 9.4 (SAS Institute).

### Role of the funding source

The funding source provided the enteral nutrition as well as the experimental treatment and the placebo. The academic authors obtained the clinical data and clinical measures. Co-authors employed by the funder participated, in collaboration with the academic co-authors, in the study design, statistical analysis, data interpretation, and review of the manuscript.

## Results

Enrolment began on 1 June 2017 and ended on 31 December 2019. Of 52 patients screened for eligibility, 35 were enrolled and randomly assigned to receive placebo (*n* = 17) or amino acids (*n* = 18) (Additional file 1: Fig. S2). Thirty-three patients (94%) were affected by sepsis, none of whom exhibited abdominal sepsis. The baseline characteristics of the patients are described in Table 1. Enteral nutrition was administered for a median of 7.5 [5, 14] days in the amino acid group compared to 10 [7; 22] days in the placebo group. During the interventional period, the total amount of energy provided by enteral nutrition was 10 956 [6 769; 25 577] kcal in the amino acid group vs. 14 090 [10 703; 34 554] kcal in the placebo group (Table 2). The daily median number of ingested calories was 20.35 [16.23; 25.35] and 22.69 [17.81; 23.87] kcal/kg/day, respectively, in the amino acid and placebo groups. The daily median amount of ingested protein was 0.97 [0.77; 1.21] and 1.08 [0.85; 1.14] g/kg/day in the amino acid and placebo groups, respectively. Thirty survivors (placebo (*n* = 15), amino acids (*n* = 15)) were followed up until day 21. Only 8 surviving patients (placebo (*n* = 4), amino acids (*n* = 4)) returned to the hospital for follow-up on day 180, and only 7 surviving patients (placebo (*n* = 3), amino acids (*n* = 4)) returned to the hospital for follow-up on day 360.

**Table 1:** Baseline characteristics

	Placebo (*n* = 17)	Amino acid (*n* = 18)
Age (years)	71 (62–75)	71 (52–83)
Male sex	12 (70.6)	9 (50)
Height (cm)	175 (163–178)	165 (160–175)
Weight (kg)	78.5 (61.9–91)	65.1 (53.5–86.6)
BMI (kg/m^2^)	26.9 (21.8–28.7)	24.2 (22–26)
Admission category		
Elective surgery	1 (5.9)	0 (0)
Emergency surgery	1 (5.9)	2 (11.1)
Medical	15 (88.2)	16 (88.9)
Main cause of admission		
Sepsis	15 (88)	18 (100)
ARDS	2 (12)	0 (0)
Source of sepsis		
Lung	13 (86.6)	14 (77.7)
Urinary tract	0 (0)	1 (5.6)
Soft tissue	1 (6.7)	2 (11.1)
Other	1 (6.7)	1 (5.6)
SOFA	10 (8–12)	9 (6–11)
SAPS II	50 (45–60)°	43 (32–71)
Vasopressor support at the time of randomization	5 (29.4)	5 (27.8)
Invasive mechanical ventilation at the time of randomization	14 (82.4)	17 (94.4)
Receipt of corticosteroids during the ICU stay	11 (64.7%)	14 (77.8%)

Data are expressed as *n* (%) or as the median (IQR)

*ARDS* acute respiratory distress syndrome, *SOFA* sequential organ failure assessment, *SAPS II* Simplified Acute Physiology Score II, *BMI* body mass index, *ICU* intensive care unit

**Table 2:** Enteral nutrition intake during the interventional period

	Placebo (*n* = 17)	Amino acid (*n* = 18)	*P*
Enteral nutrition administration (days)	10 (7–22)	7.5 (5–14)	0.39
Between trial day 1 and trial day 7 (kcal)	11 336 (7 839–13 676)	8 621 (6 769–11 940)	0.20
Between trial day 8 and trial day 14 (kcal)	10 088 (3 007–11 558)	8 522 (3 481–12 490)	0.88
Between trial day 15 and trial day 21 (kcal)	11 522 (11 085–14 204)	9 126 (5 232–12 542)	0.36

Data are expressed as the median (IQR)

Wilcoxon two-sample test

### Effects on skeletal muscles and diaphragm

**Fig. 1:**
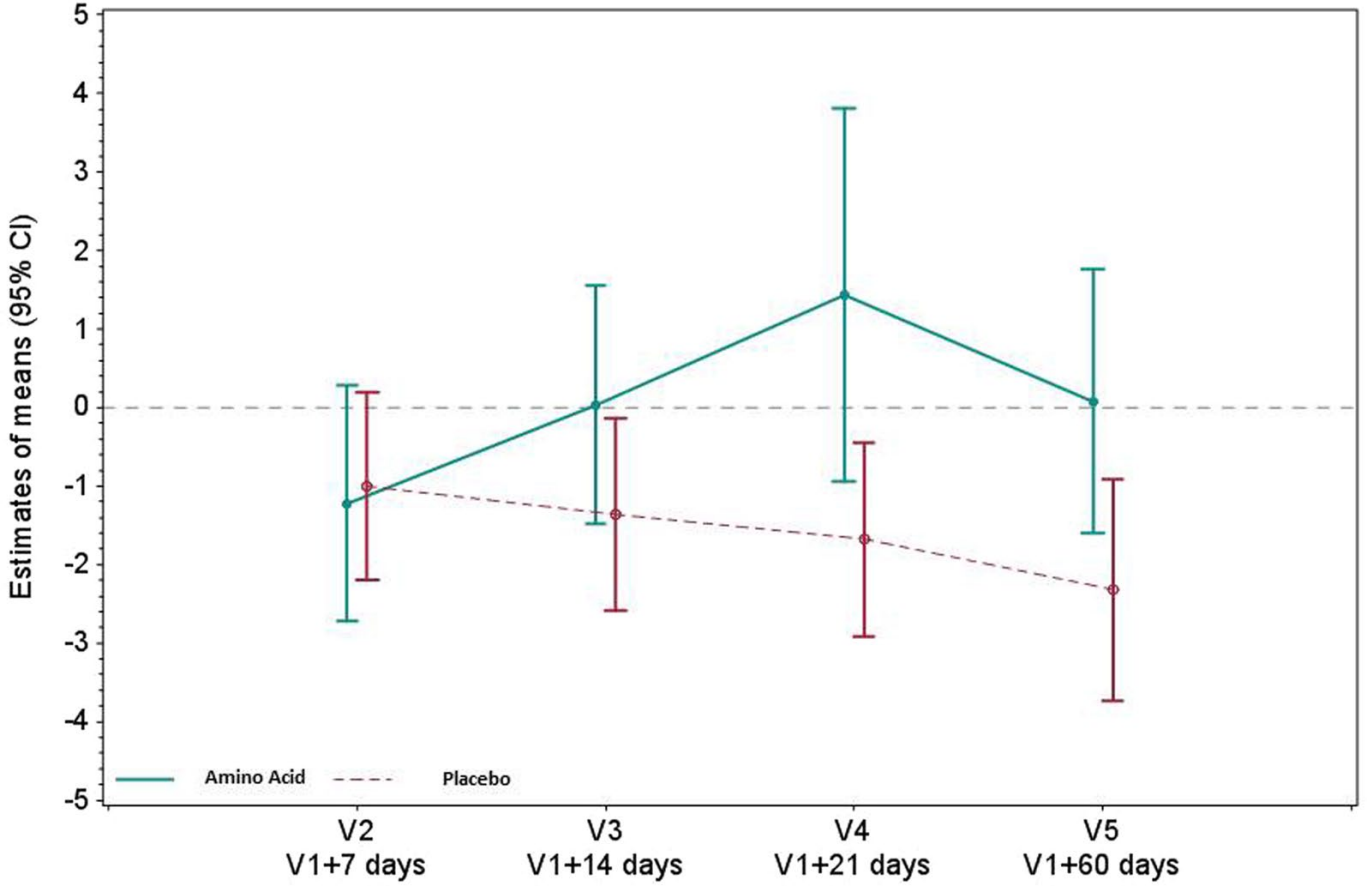
Indexed muscle volume of the anterior compartment of the quadriceps (cc/kg) in the amino acid and placebo groups. Anterior quadriceps volume was measured semi-automatically using isometric T1-weighted 3D images. Data are presented as the mean value of both legs. Muscle volume was indexed to the body weight. Values reported are mean changes from baseline at days 7, 14, 21 and 60, using a restricted maximum likelihood (REML)-based repeated measures approach. *P* < 0.05 at V4 and at V5

**Fig. 2:**
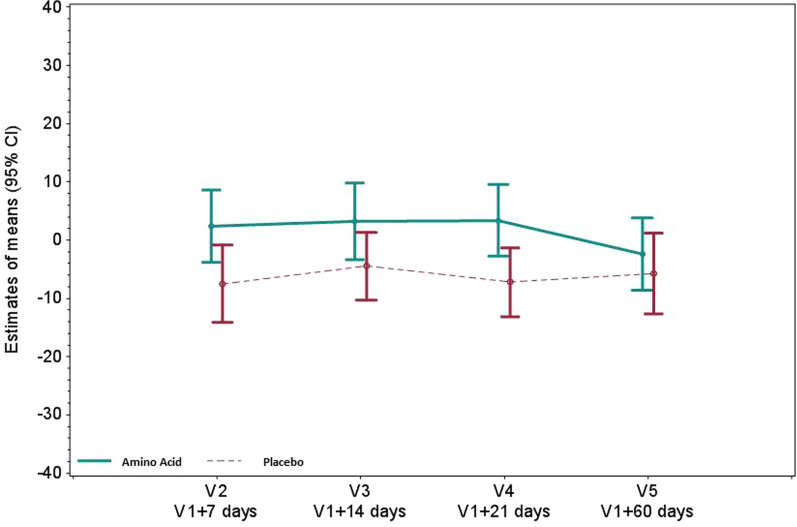
Twitch airway pressure (cm H_2_O) in the amino acid and placebo groups. Twitch airway pressure was measured following magnetic stimulation over the spinal processes between C5 and C7. Values reported are mean changes from baseline at days 7, 14, 21 and 60, using a restricted maximum likelihood (REML)-based repeated measures approach. *P* < 0.05 at V4

Relative to baseline, the indexed muscle volume of the anterior compartment of the quadriceps was significantly greater in the amino acid group at 3 weeks (difference in means compared to baseline [95% CI] 3.12 [0.5; 5.73] cm^3^/kg; *P* = 0.022) (Fig. 1, Additional file 1: Table S1). Leg muscle strength was not compared between groups due to the amount of missing data (Additional file 1: Table S2). Relative to baseline, the twitch airway pressure increased up to 3 weeks after randomization in the amino acid group compared to the placebo group. Three weeks after randomization, the twitch airway pressure relative to baseline was significantly greater in the amino acid group than in the placebo group (difference in means compared to baseline [95% CI] 10.6 [0.99; 20.20] cmH_2_0; *P* = 0.035) (Fig. 2, Additional file 1: Table S3). Forced vital capacity did not differ between groups, overall (difference in means relative to baseline [95% CI] − 0.31 [− 0.89; 0.27] L; *P* = 0.265) (Additional file 1: Table S4). Urine 3-methyl histidine, a marker of muscle protein catabolism, decreased similarly in both groups overall, (difference in means relative to baseline values [95% CI] 0.13 [− 1.27; 1.02] μmol/kg/day; *P* = 0.827) (Additional file 1: Table S5).

### Effects on gut functionality

**Fig. 3:**
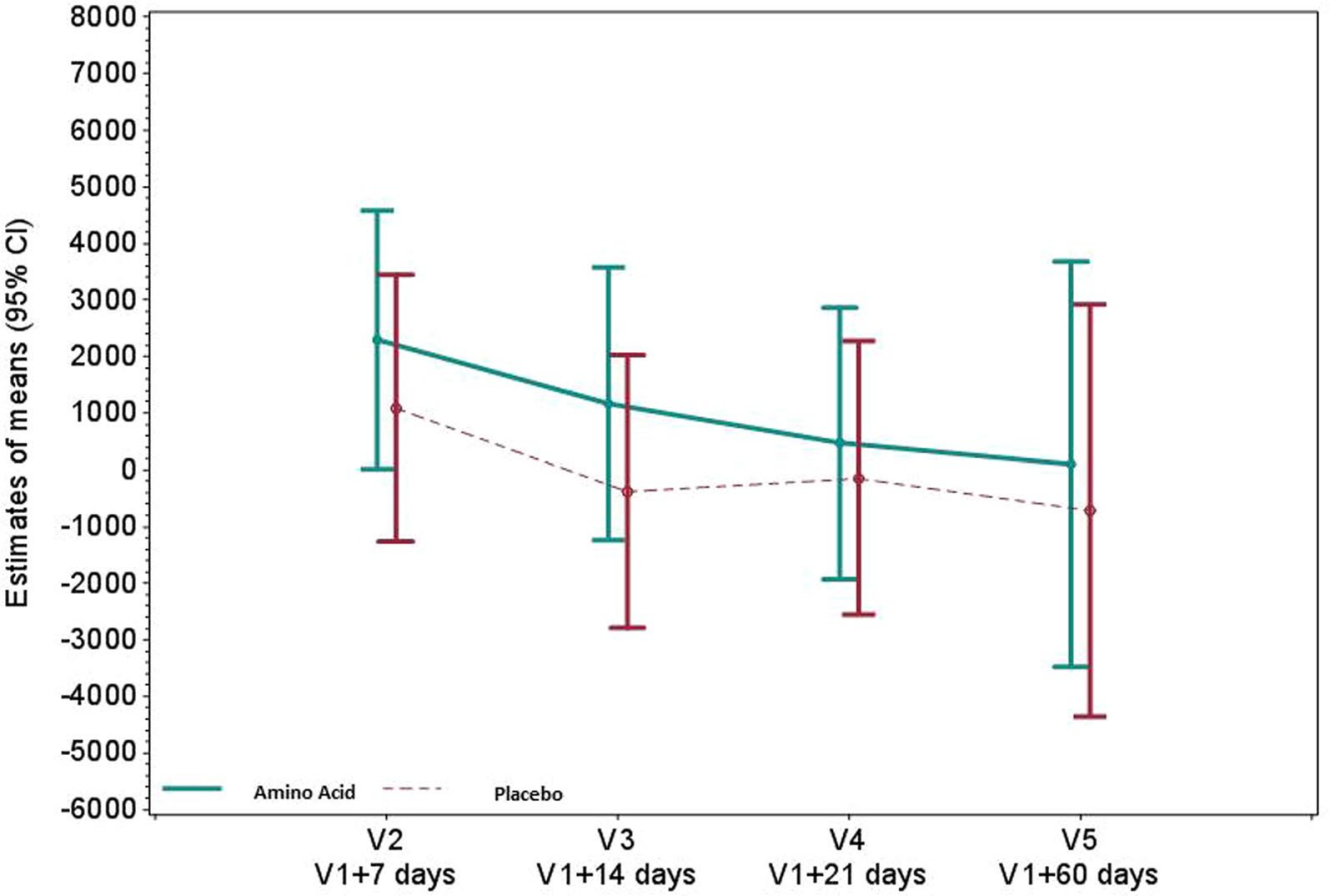
Plasma I-FABP (pg/mL) in the amino acid and placebo groups. Plasma I-FABP was assessed by ELISA. Values reported are mean changes from baseline at days 7, 14, 21 and 60, using a restricted maximum likelihood (REML)-based repeated measures approach

**Fig. 4:**
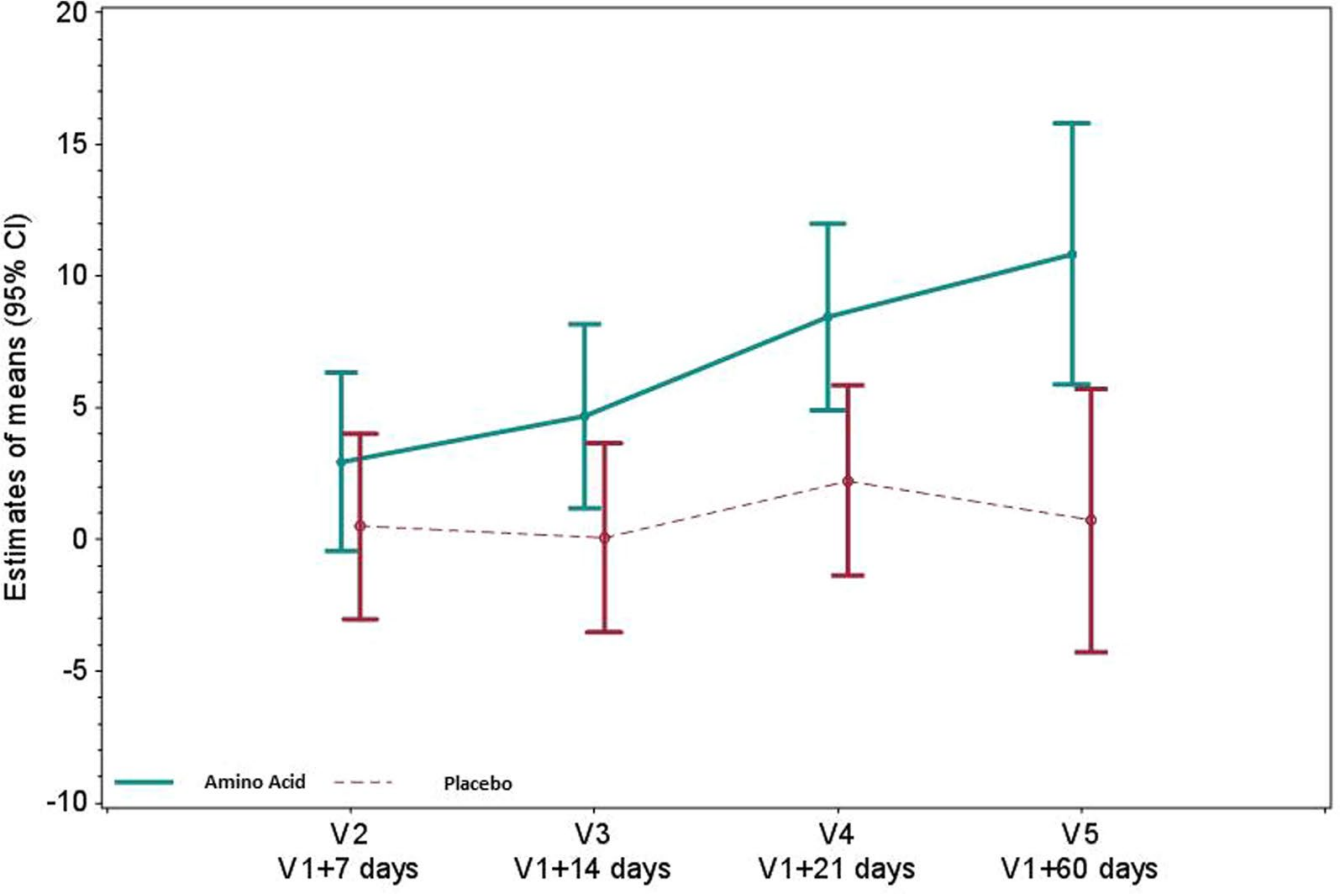
Plasma citrulline (nmol/mL) in the amino acid and placebo groups. Plasma citrulline concentration was assessed by ion exchange chromatography with spectrophotometric detection after ninhydrin derivatization. Values reported are mean changes from baseline at days 7, 14, 21 and 60, using a restricted maximum likelihood (REML)-based repeated measures approach. *P* < 0.05 at V4 and *P* < 0.01 at V5

Plasma intestinal fatty acid-binding protein (I-FABP), a marker of enterocyte injury, did not differ between groups (ratio of geometric means relative to baseline [95% CI] 1.23 [0.77; 1.96] *P* = 0.379) (Fig. 3, Additional file 1: Table S6). Plasma citrulline, a marker of bioactive enterocyte mass, significantly increased in the amino acid group compared to the placebo group with a difference in means relative to baseline [95% CI] of 5.86 [1.72; 10.00] nmol/mL (*P* = 0.007) (Fig. 4, Additional file 1: Table S7).

### Effects on inflammation and hepatic function

The overall effect of amino acid supplementation on the kinetics of acute phase proteins C-reactive protein, procalcitonin, ferritin and fibrinogen (Additional file 1: Tables S8–S11) was not significant. Faecal calprotectin concentration was similar between groups, with a difference in means relative to baseline [95% CI] of − 170.58 [− 461.37; 120.20] μg/g (*P* = 0.217) (Additional file 1: Table S12).

**Fig. 5:**
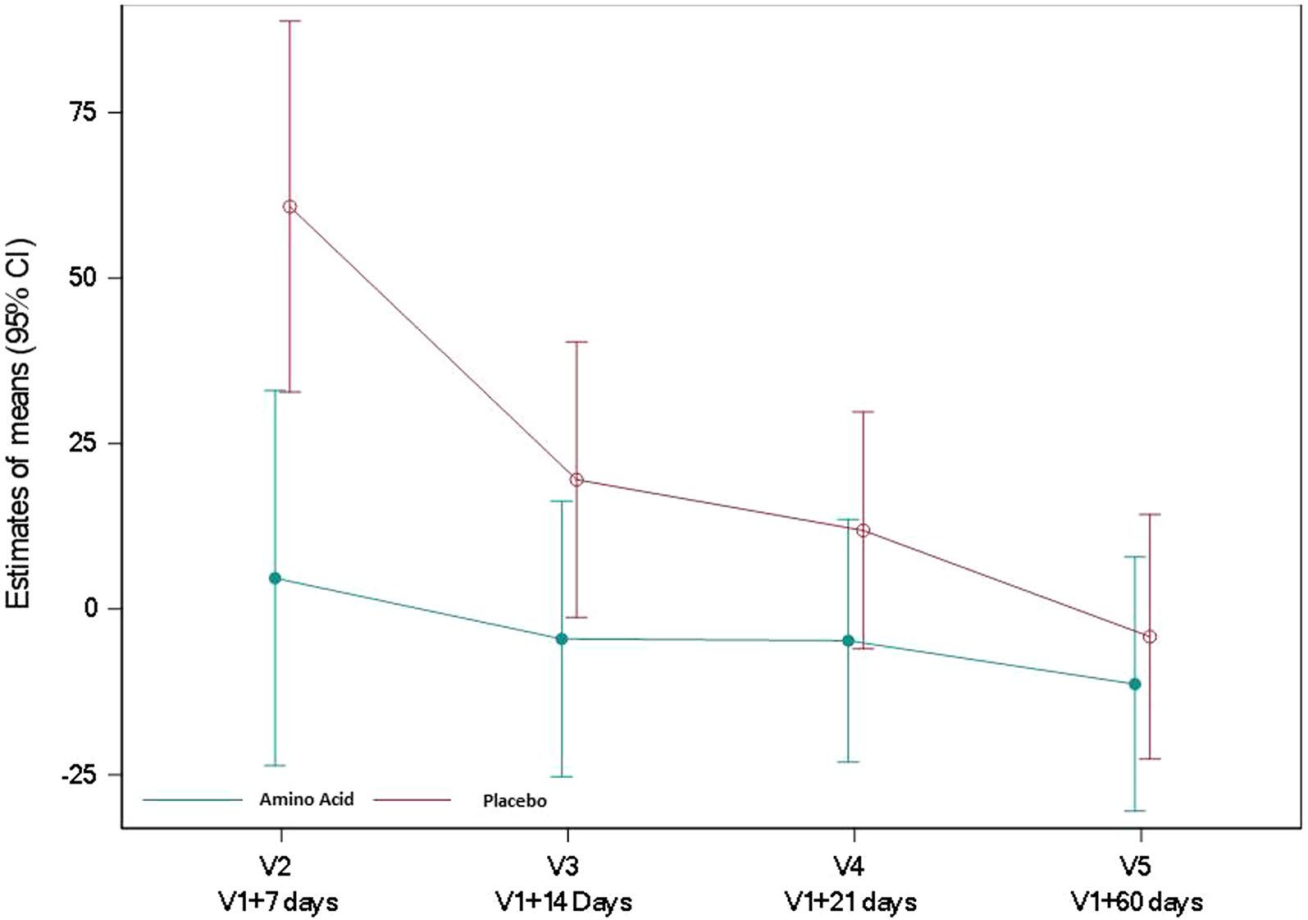
Alanine aminotransferase (UI/L) in the amino acid and placebo groups. Alanine aminotransferase concentration was assessed by spectrophotometry. Values reported are mean changes from baseline at days 7, 14, 21 and 60, using a restricted maximum likelihood (REML)-based repeated measures approach. *P* < 0.05 at V2

**Fig. 6:**
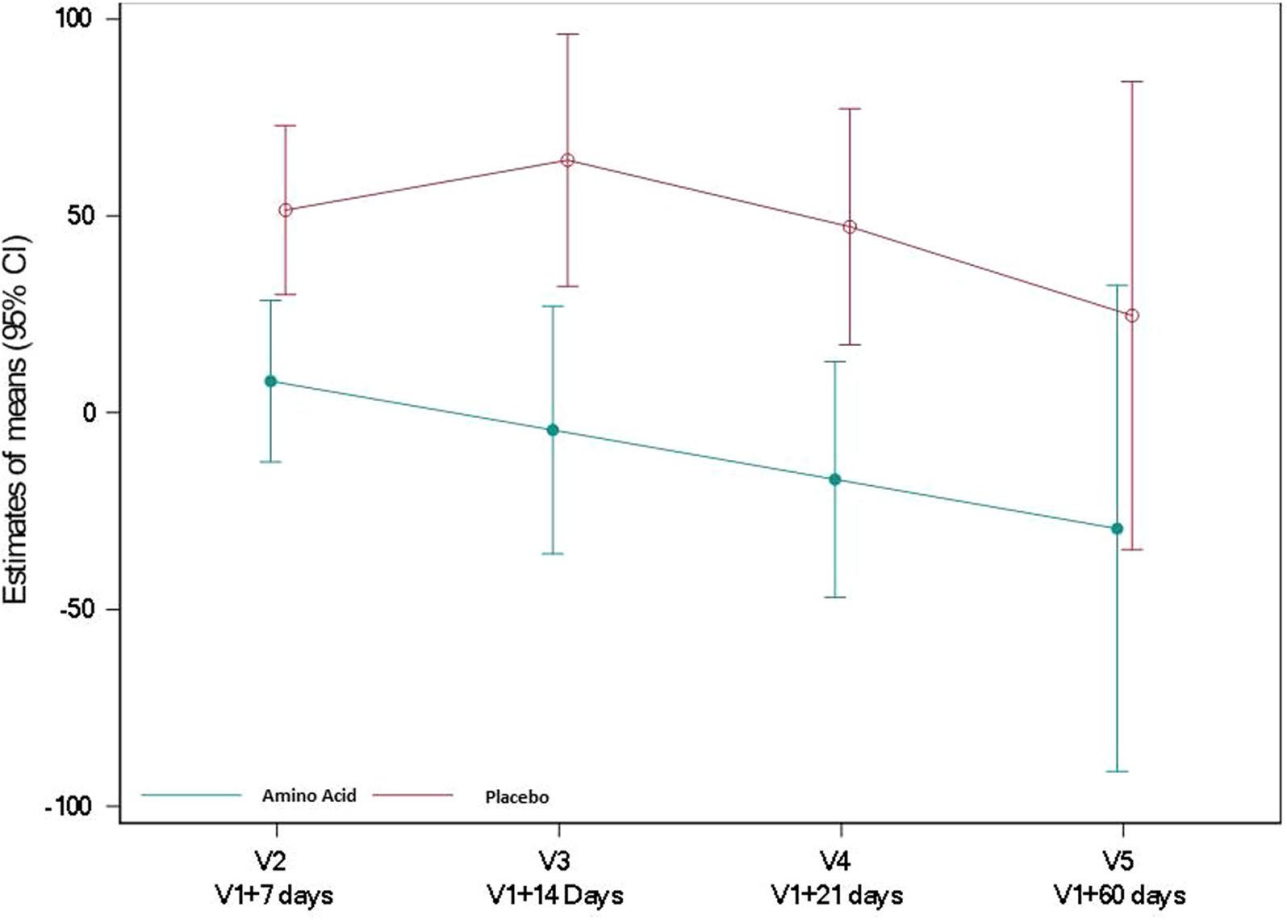
Alkaline phosphatase (UI/L) in the amino acid and placebo groups. Alkaline phosphatase concentration was assessed by spectrophotometry. Values reported are mean changes from baseline at days 7, 14, 21 and 60, using a restricted maximum likelihood (REML)-based repeated measures approach. *P* < 0.02 at V2, *P* < 0.001 at V3, *P* < 0.002 at V4

**Fig. 7:**
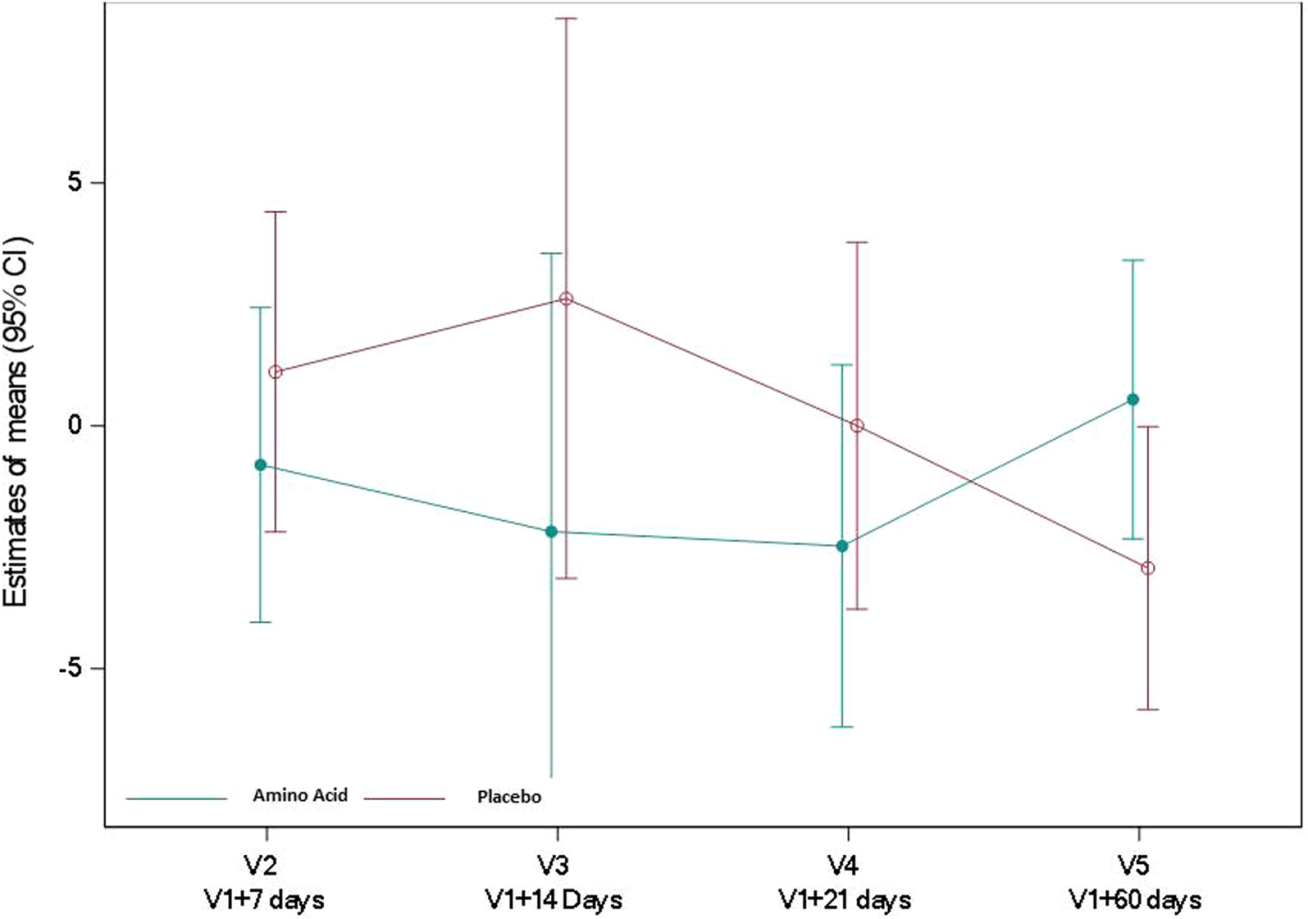
Total bilirubin (µmol/L) in the amino acid and placebo groups. Total bilirubin concentration was assessed by spectrophotometry. Values reported are mean changes from baseline at days 7, 14, 21 and 60, using a restricted maximum likelihood (REML)-based repeated measures approach

Relative to baseline, alanine aminotransferase concentration was lower in the amino acid group than in the placebo group at one week with a ratio of geometric means relative to baseline [95% CI] of 0.5 [0.29; 0.86] (*P* = 0.015) (Fig. 5, Additional file 1: Table S13). Relative to baseline, alkaline phosphatase concentration was lower in the amino acid group than in the placebo group, with an overall ratio of geometric means relative to baseline [95% CI] of 0.67 [0.52; 0.88] (*P* = 0.005) (Fig. 6, Additional file 1: Table S14). Bilirubin levels did not significantly differ between groups, with a ratio of geometric means relative to baseline [95% CI] of 0.89 [0.61; 1.3] (*P* = 0.531) (Fig. 7, Additional file 1: Table S15). Prealbumin increased over time in both groups, with a difference in means relative to baseline values [95% CI] of 0.03 [− 0.02; 0.08] g/L (*P* = 0.206) (Additional file 1: Table S16). Albumin increased over time in both groups, with a difference in means relative to baseline values [95% CI] of 0.97 [− 2.89; 4.82] g/L (*P* = 0.611) (Additional file 1: Table S17).

### Amino acid concentration and nutrient profile

Overall, plasma levels of amino acids were low at baseline in both groups. The investigational treatment significantly increased the plasma concentrations of threonine with a difference in means relative to baseline of 51.33 [13.33; 89.33] nmol/ml *P* = 0.009; proline with a difference in means relative to baseline of 46.53 [5.20; 87.86] nmol/L *P* = 0.028; serine with a difference in means relative to baseline of 13.33 [0.66; 26.01] nmol/ml *P* = 0.04; and cysteine with a difference in means relative to baseline of 10.69 [1.47; 19.91] nmol/mL *P* = 0.024. Leucine levels only significantly increased on day 7 after randomization in the amino acid group with a difference in means relative to baseline of 32.18 [6.16; 58.21] nmol/mL *P* = 0.016, (Additional file 1: Tables S18–S22). Increased plasma levels of threonine, proline, serine, cysteine and leucine demonstrate treatment compliance. Specific amino acid concentration subsequently decreased on days 21 and 60, caused by the resumption of oral feeding in most patients. The plasma concentrations of isoleucine, lysine, methionine, phenylalanine, tryptophan, valine, arginine, asparagine, tyrosine, histidine, aspartic acid, glutamic acid, glycine, taurine and ornithine did not significantly differ between groups. Plasma concentration of arginine, difference in means relative to baseline of 8.40 [− 3.21; 20.02] nmol/ml *P* = 0.15 and of glutamine 33.00 [− 18.60; 84.60] nmol/ml *P* = 0.203 did not differ between groups. However, plasma levels of arginine decreased until visit 3 in the placebo group, and plasma levels of glutamine decreased until visit 2 in the placebo group, while plasma levels of arginine and glutamine increased over time in the amino acid group (Additional file 1: Tables S23 and S24). Zinc and cholesterol levels (Additional file 1: Tables S25 and S26) were higher in the amino acid group at one week of treatment (ratio of geometric means relative to baseline [95% CI] 1.14 [1; 1.29] *P* = 0.042 and 1.26 [1.07; 1.49] *P* = 0.007, respectively).

### Clinical outcomes

The number of ICU-free days until day 21 was 9.5 [0; 13] days in the amino acid group vs. 6.0 [0; 12] in the placebo group (*P* = 0.457) (Additional file 1: Fig. S1). The number of ventilation-free days until day 21 was 12.5 [4; 18] days in the amino acid group vs. 10 [0; 17] days in the placebo group (*P* = 0.316). Sixty days after randomization, 8/13 (61.5%) patients were walking in the amino acid group versus 5/12 (31.7%) in the placebo group (*P* = 0.434).

### Safety

The experimental product was well tolerated with no evidence of renal impairment based on urine output, serum creatinine level and glomerular filtration rate (Table 3). Specifically, the administration of amino acids was not temporally associated with any evidence of oliguria or kidney injury. Amino acid supplementation induced a transient increase in the related amino acid concentration without ever reaching potentially toxic plasma concentrations. Nine deaths occurred during the trial, of which 3 deaths occurred in the amino acid group vs. 6 deaths in the placebo group. No death was related to the administration of the study product.

**Table 3:** Renal parameters

	Placebo	Amino acid
	*N* = 17	*N* = 18
*V1 (Baseline)*		
Urine output (mL/24 h)	1300 (950–2500)	1700 (500–2350)
Serum creatinine (µmol/L)	70 (54–120)	63 (51–92)
Glomerular filtration rate (mL/min/1.73 m^2^)	91 (49–105)	90 (64–115)
*V2 (V1* + *7 days)*		
Urine output (mL/24 h)	1300 (900–2100)	1050 (800–2300)
Serum creatinine (µmol/L)	72 (48–94)	55.5 (47.5–71)
Glomerular filtration rate (mL/min/1.73 m^2^)	93.5 (65.5–107.5)	101 (82.5–116)
*V3 (V1* + *14 days)*		
Urine output (mL/24 h)	1375 (625–2450)	950 (725–1975)
Serum creatinine (µmol/L)	72 (51–98)	57 (48–92)
Glomerular filtration rate (mL/min/1.73 m^2^)	92 (64–110)	93 (71–112)
*V4 (V1* + *21 days)*		
Urine output (mL/24 h)	1650 (1000–2000)	900 (700–1500)
Serum creatinine (µmol/L)	63 (48–84)	61 (37–82)
Glomerular filtration rate (mL/min/1.73 m^2^)	90 (77–108)	101 (78–107)
*V5 (V1* + *60 days)*		
Urine output (mL/24 h)	1800 (1600–2000)	900 (400–5050)
Serum creatinine (µmol/L)	52.5 (51–81)	66.5 (50–71)
Glomerular filtration rate (mL/min/1.73 m^2^)	99 (86–109)	101 (82–104)

Data are expressed as the median (IQR)

## Discussion

In this randomized controlled, double-blind trial, enteral supplementation by a specifically tailored blend of threonine, cysteine, proline, serine and leucine did not alter renal function as measured by creatinine or urine output. Moreover, amino acid supplementation significantly increased the corresponding amino acid plasma concentration without ever reaching toxic levels. The amino acid blend assessed in the current trial can therefore be considered safe in septic and ARDS patients.

We consequently sought to describe the physiological consequences of amino acid supplementation. Bacterial translocation is associated with infectious complications and promotes systemic inflammation [48]. Maintenance of a functional gut barrier function depends partly on the mucus outer layer, which contains secreted gel-forming mucins. Intestinal mucins contain large quantities of threonine, serine, proline and cysteine. Optimal concentrations of these four amino acids are therefore central to maintaining an effective intestinal barrier function. Previous studies in animal models demonstrated that administrating a blend of threonine, serine, cysteine and proline increased mucin synthesis during intestinal injury [20, 21]. We observed high plasma I-FABP levels in septic patients, indicating enterocyte injury. In contrast, supplementation by amino acids increased the citrulline concentration. These findings indicate that the amino acid blend increases the functional enterocyte mass and thus may accelerate gut healing and recovery of gut barrier function. Improved gut barrier function may reduce systemic exposure to pathogens and thereby help resolve inflammation.

Critically ill patients, especially those with long ICU stays, exhibit significant muscle mass loss. Indeed muscles act as amino acid reservoirs, providing amino acids to the splanchnic area for anabolism. In this context, it is of particular interest to observe that the present amino acid blend increased the quadriceps volume and improved the diaphragm muscle strength, suggesting that muscle catabolism to supply free amino acids is decreased when enough amino acids were supplied. Physical rehabilitation may also contribute to reducing muscle weakness. Combining higher protein input and physical rehabilitation needs to be assessed in randomized controlled trials [12].

Observational studies indicate that decreased protein intake is associated with worse clinical outcomes in critically ill patients [13, 14]. However, in previous trials, the administration of higher doses of a balanced set of amino acids failed to improve outcomes in the critically ill [49, 50]. These trials may have failed to demonstrate a clinical benefit because the wrong dose, the wrong route of administration, or the wrong amino acid composition was assessed. Indeed, single amino acid supplementation worsens outcomes in the critically ill. Supplementation by glutamine in the critically ill was not associated with an improved outcome and was even associated with an increased risk of death in the sickest patients [51, 52]. Supplementation with arginine may also be associated with an increased risk of death in the sickest patients [53].

Administrating a specially tailored formula of amino acids may be a better way of improving clinical outcomes. Supplementation by serine, threonine, cysteine and proline directly meets the nutritional requirement of the splanchnic area (gut and liver), thereby decreasing muscle protein breakdown. Threonine plays a major role in the maintenance of gut barrier function [19, 20]. Supplementation by cysteine, which is necessary for the synthesis of glutathione, a major hepatic antioxidant, may be responsible for the observed improvement in liver function in amino acid-treated patients. However, we did not measure antioxidant activity in our patients, meaning that we could not formally prove this hypothesis. Leucine plays a role in the increase in muscle protein synthesis. However, leucine, while inefficient during acute inflammation, stimulates protein synthesis during the recovery phase. A previous study in trauma patients showed that the administration of amino acids, including threonine, serine, cysteine and aspartate, targeting gut and liver anabolic requirements translated into increased glutathione and muscle protein synthesis [54]. An important finding of the current trial is that the plasma levels of glutamine and arginine, both considered conditionally essential amino acids in critical illness, did not drop in the supplemented group, suggesting that supplemented amino acids can sustain endogenous synthesis of these amino acids, leucine and proline being their respective precursors [55–57].

Last, the total amount of administered calories was larger in the placebo group than in the amino acid group. When considering the daily number of administered calories indexed to the weight of patients, this difference disappeared, explained in part by the fact that patients in the placebo group were ventilated (and therefore received enteral nutrition) a median of 2 days longer than patients in the amino acid group. The observed caloric differences between groups may also be explained by the slight gender imbalance between groups. More males were randomized to the placebo group, leading to slightly skewed anthropometrics (i.e. greater body weight in the placebo group). These points may, therefore, be confounding factors.

Our study has several strengths: the treatment was randomly assigned and compared to placebo, and the patients and investigators were all blinded to the allocated treatment. We performed an extensive physiological exploration of highly relevant muscle- and gut-related parameters in the critically ill; a functional assessment of diaphragm and quadriceps muscle strength was assessed in a nonvolitional manner, limiting the risk of bias.

Our study has several limitations, including the small size of our population. The size was dictated by the thorough clinical, biological, functional and radiological explorations performed in the target patient population. Consequently, we observed some level of gender imbalance in the placebo group, with only 29.4% female patients in that group versus 50% female patients in the amino acid group. This gender imbalance was associated with slightly skewed anthropometrics, height, weight and BMI being greater in the placebo group. We sought to control for this factor by including sex as a covariate in the statistical model. Due in part to difficulties in predicting the ICU course of patients at the time of admission, enteral feeding was not administered for the full 3-week period as initially planned. This may have reduced the effectiveness of the experimental product. We also reported missing data in several functional assessments. These assessments were not always performed during the acute phase because patients were too sick for mobilization. Moreover, during follow-up, several patients declined to perform subsequent nonvolitional muscle strength assessments due to the perceived discomfort associated with the technique.

## Conclusion

A specific blend of five amino acids (threonine, cysteine, proline, serine and leucine) added to the enteral nutrition of ICU patients with sepsis or ARDS was safe and was associated with improved skeletal muscle, diaphragm and gut functions. These findings provide a rationale for further clinical investigations.

## Supplementary Information

**Additional file 1. Supplemental Figures and Tables. Supplemental Figure 1:** Study design. **Supplemental Figure 2**: Flow chart. **Supplemental Table 1**: Indexed muscle volume of the anterior compartment of the quadriceps. **Supplemental Table 2**: Muscle Strength. **Supplemental Table 3**: Twitch airway pressure. **Supplemental Table 4**: Forced vital capacity. **Supplemental Table 5**: Urine 3 methyl-histidine. **Supplemental Table 6**: Plasma I-FABP. **Supplemental Table 7**: Plasma citrulline. **Supplemental Table 8**: C-reactive protein. **Supplemental Table 9**: Procalcitonin. **Supplemental Table 10**: Ferritin. **Supplemental Table 11**: Fibrinogen. **Supplemental Table 12**: Feacal calprotectin. **Supplemental Table 13**: Alanine aminotransferase. **Supplemental Table 14**: Alkaline phosphatase. **Supplemental Table 15**: Bilirubin. **Supplemental Table 16**: Pre-albumin. **Supplemental Table 17**: Albumin. **Supplemental Table 18**: Threonine **Supplemental Table 19**: Proline. **Supplemental Table 20**: Serine. **Supplemental Table 21**: Cysteine. **Supplemental Table 22**: Leucine. **Supplemental Table 23**: Arginine. **Supplemental Table 24**: Glutamine. **Supplemental Table 25**: Zinc. **Supplemental Table 26**: Cholesterol.

## Data Availability

Data sharing requests will be considered by the management group upon written request to the corresponding author. Deidentified participant data or other prespecified data may be made available subject to a written proposal and a signed data sharing agreement.
