# Supplementary material for: Effect of an enteral amino acid blend on muscle and gut functionality in critically ill patients: a proof-of-concept randomized controlled trial
Source: Crit Care. 2022 Nov 17;26:358. doi: 10.1186/s13054-022-04232-5 (PMC9670468; doi:10.1186/s13054-022-04232-5)
Supplement: Supplementary file 1 — Additional file 1. Supplemental Figures and Tables. Supplemental Figure 1: Study design. Supplemental Figure 2: Flow chart. Supplemental Table 1: Indexed muscle volume of the anterior compartment of the quadriceps. Supplemental Table 2: Muscle Strength. Supplemental Table 3: Twitch airway pressure. Supplemental Table 4: Forced vital capacity. Supplemental Table 5: Urine 3 methyl-histidine. Supplemental Table 6: Plasma I-FABP. Supplemental Table 7: Plasma citrulline. Supplemental Table 8: C-reactive protein. Supplemental Table 9: Procalcitonin. Supplemental Table 10: Ferritin. Supplemental Table 11: Fibrinogen. Supplemental Table 12: Feacal calprotectin. Supplemental Table 13: Alanine aminotransferase. Supplemental Table 14: Alkaline phosphatase. Supplemental Table 15: Bilirubin. Supplemental Table 16: Pre-albumin. Supplemental Table 17: Albumin. Supplemental Table 18: Threonine Supplemental Table 19: Proline. Supplemental Table 20: Serine. Supplemental Table 21: Cysteine. Supplemental Table 22: Leucine. Supplemental Table 23: Arginine. Supplemental Table 24: Glutamine. Supplemental Table 25: Zinc. Supplemental Table 26: Cholesterol. [file 13054_2022_4232_MOESM1_ESM.docx]

**Supplemental Figures and Tables**

**Effect of an enteral amino acid blend on muscle and gut functionality in critically ill patients: A proof-of-concept randomized controlled trial.**

Nicholas Heming, MD ^1,2,3,4^; Robert Carlier, MD ^5^; Helene Prigent, MD ^6^; Ahmed Mekki, MD ^5^; Camille Jousset, MD ^5^; Frederic Lofaso, MD ^6^ ; Xavier Ambrosi, MD ^1,7^; Rania Bounab, MD ^1^; Virginie Maxime, MD ^1^; Arnaud Mansart, PhD ^2,3,4^; Pascal Crenn, MD ^8^; Pierre Moine, MD ^1,2,3,4^; Fabien Foltzer, MSc^9^, Bernard Cuenoud PhD ^10^, Maurice Beaumont, MD^9^; Claudia Roessle, PhD ^10^; Mickaël Hartweg, MSc ^9^; Jean-Charles Preiser, MD ^11^;Denis Breuillé, PhD ^9^; Djillali Annane, MD ^1,2,3,4^

1- Department of Intensive Care, Hôpital Raymond Poincaré, APHP University Versailles Saint Quentin - University Paris Saclay, France.

2- Laboratory of Infection & Inflammation - U1173, School of Medicine Simone Veil, University Versailles Saint Quentin - University Paris Saclay, INSERM, Garches, France.

3- FHU SEPSIS (Saclay and Paris Seine Nord Endeavour to PerSonalize Interventions for Sepsis), Garches 92380, France

4- RHU RECORDS (Rapid rEcognition of CORticosteroiD resistant or sensitive Sepsis), Garches 92380, France

5- Department of Radiology, APHP, Hôpital Raymond Poincaré, DMU Smart Imaging, GH Université Paris-Saclay, UFR des Sciences de la Santé Simone-Veil, Université de Versailles Saint-Quentin-en-Yvelines, Montigny-le-Bretonneux, France

6- Department of Physiology-AP-HP, Hôpital Raymond-Poincaré, Garches, France; UFR des Sciences de la Santé Simone-Veil, Université de Versailles Saint-Quentin-en-Yvelines, Montigny-le-Bretonneux, France.

7-Department of Anesthesiology and Intensive Care Medicine, University Hospital of Nantes, Nantes, France

8- Clinical nutrition unit and FHU Hepatinov, Hôpital Raymond Poincaré, APHP Université Paris Saclay/UFR Simone Veil‐Santé‐Université de Versailles Saint Quentin en Yvelines, Garches, France

9- Nestlé Research, Société de Produits de Nestlé, Lausanne Switzerland

10- Translation Research, Nestlé Health Science, Lausanne Switzerland

11-Nutrition Team, Erasme University Hospital, Université Libre de Bruxelles, B-1070, Brussels, Belgium

Corresponding author:

Pr Djillali Annane, MD, PhD

General Intensive Care Unit

Raymond Poincaré Hospital (AP-HP),

University of Versailles Saint-Quentin en Yvelines

104, Boulevard Raymond Poincaré

92380 Garches France

Tel: 00 33 (0)1 47 10 77 80; Fax: 00 33 (0)1 47 10 77 83;

Email: [djillali.annane@aphp.fr](mailto:djillali.annane@aphp.fr)

Supplemental Figure 1 : Study design


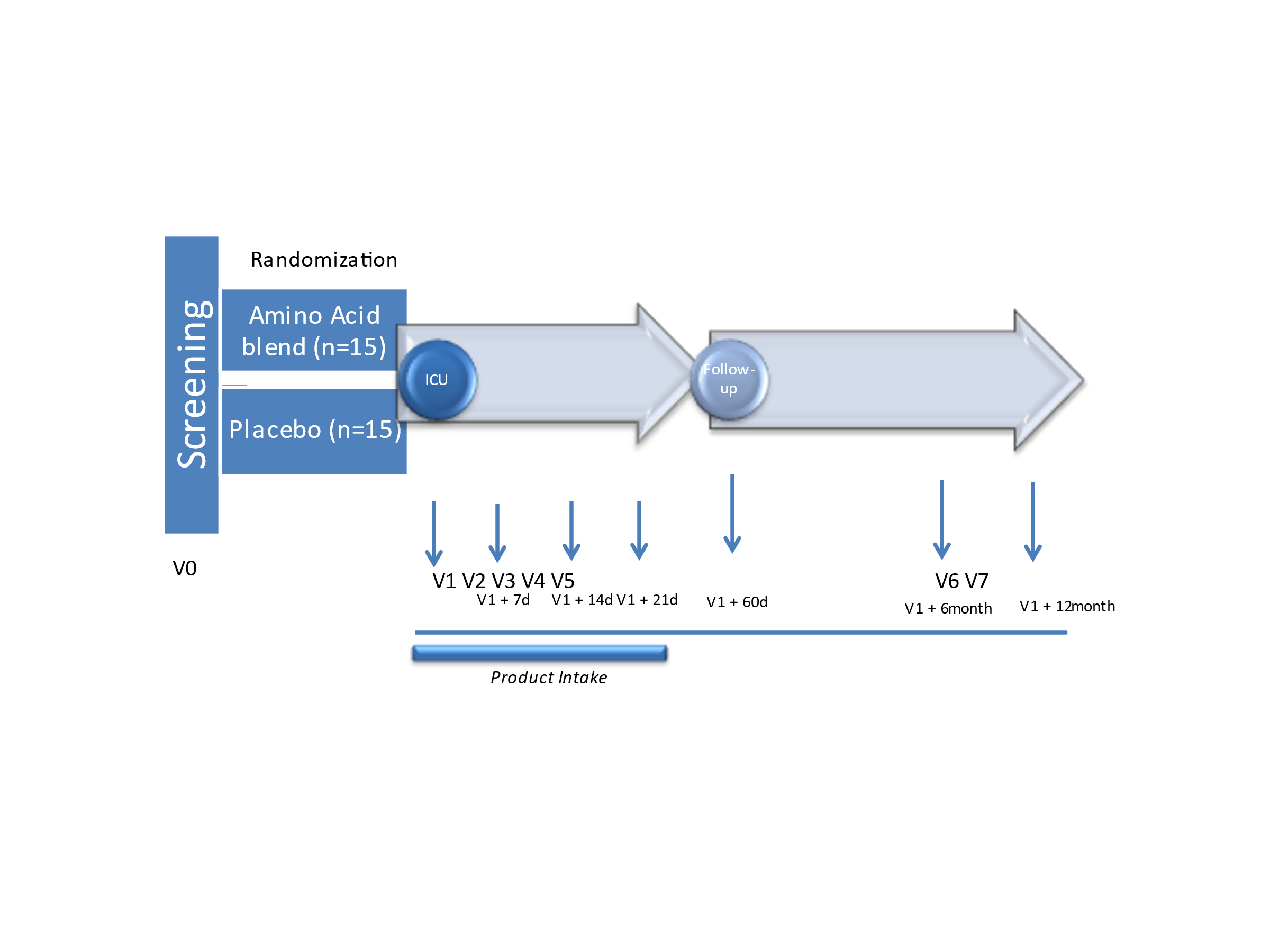


Supplemental Figure 2: Flow chart


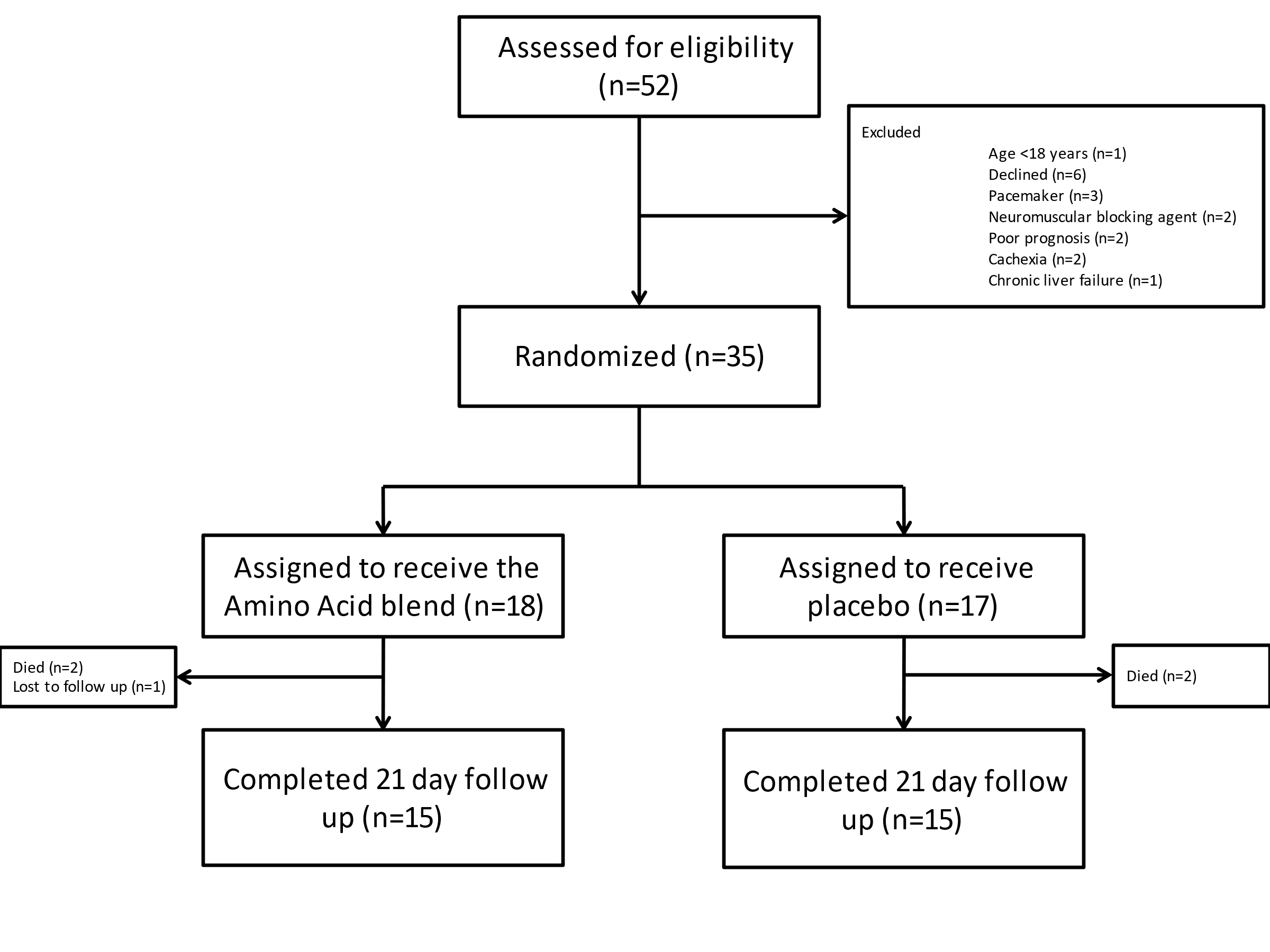


Supplemental Table 1: Indexed muscle volume of the anterior compartment of the quadriceps (cm^3^/kg, mean of both legs)

| Visit | Variable | Placebo N=17 | Amino Acid N=18 |
| --- | --- | --- | --- |
| V1 (Baseline) | n | 14 | 10 |
|  | Indexed muscle volume anterior compartment of the quadriceps (cm3/kg) | 9.64 [7.60; 11.56]  9.90 (± 2.55) | 7.91 [6.75; 9.71]  8.53 (± 2.96) |
|  |  |  |  |
| V2 (V1+7 days) | n | 11 | 9 |
|  | Indexed muscle volume anterior compartment of the quadriceps (cm3/kg) | 8.39 [6.96; 12.20]  9.11 (± 3.09) | 7.46 [6.80; 9.37]  8.97 (± 3.70) |
|  |  |  |  |
| V3 (V1+14 days) | n | 8 | 4 |
|  | Indexed muscle volume anterior compartment of the quadriceps (cm3/kg) | 7.58 [6.74; 8.88]  7.98 (± 2.19) | 9.27 [7.48; 13.23]  10.36 (± 4.28) |
|  |  |  |  |
| V4 (V1+21 days) | n | 8 | 3 |
|  | Indexed muscle volume anterior compartment of the quadriceps (cm3/kg) | 9.69 [7.60; 12.62]  9.95 (± 3.14) | 5.80 [3.85; 17.23]  8.96 (± 7.23) |
|  |  |  |  |
| V5 (V1+60 days) | n | 5 | 4 |
|  | Indexed muscle volume anterior compartment of the quadriceps (cm3/kg) | 5.59 [5.47; 11.41]  8.14 (± 3.96) | 9.23 [6.60; 13.26]  9.93 (± 4.63) |

Data are expressed as the median [IQR], and mean (±SD)

Muscle volume was indexed to the weight, obtained on the same day as MRI was performed.

Data are missing between V1 and V4 because patients were too sick to be moved

Supplemental Table 2: Muscle Strength (Newton, Mean of both legs)

| Visit | Variable | Placebo N=17 | Amino Acid N=18 |
| --- | --- | --- | --- |
| V1 (Baseline) | n | 8 | 7 |
|  | Muscle strength (N) | 38.2 [22.6; 59.4]  41.00 (± 23.57) | 21.2 [18.2; 67.7]  39.25 (± 35.04) |
| V2 (V1+7 days) | n | 8 | 9 |
|  | Muscle strength (N) | 32.6 [19.9; 66]  48.38 (± 40.72) | 20.5 [18.5; 39.2]  41.67 (± 41.85) |
| V3 (V1+14 days) | n | 12 | 8 |
|  | Muscle strength (N) | 41.5 [25.9; 59] 42.25 (± 23.79) | 16.5 [13.2; 33.7]  33.94 (± 42.23) |
| V4 (V1+21 days) | n | 10 | 8 |
|  | Muscle strength (N) | 42.9 [28.5; 59.7] 49.38 (± 30.57) | 36.9 [21.1; 49]  42.13 (± 30.65) |
| V5 (V1+60 days) | n | 5 | 4 |
|  | Muscle strength (N) | 70.7 [6; 83.5] 57.30 (± 50.95) | 56.4 [38.4; 90.9]  64.63 (± 38.37) |

Data are expressed as the median [IQR], and mean (±SD)

Data are missing between V1 and V4 because patients were too sick to be moved

Supplemental Table 3: Twitch airway pressure (cmH_2_O)

| Visit | Variable | Placebo N=17 | Amino Acid N=18 |
| --- | --- | --- | --- |
| V1 (Baseline) | n | 7 | 5 |
|  | Twitch airway pressure (cmH2O) | 9 [6.6; 13]  10.37 (± 6.15) | 17 [9; 18]  14.80 (± 11.17) |
|  |  |  |  |
| V2 (V1+7 days) | n | 6 | 9 |
|  | Twitch airway pressure (cmH2O) | 12.5 [10; 14]  12.17 (± 2.48) | 13 [10; 14]  11.89 (± 3.62) |
|  |  |  |  |
| V3 (V1+14 days) | n | 9 | 5 |
|  | Twitch airway pressure (cmH2O) | 13 [7; 16]  12.42 (± 8.04) | 10 [10; 13]  11.90 (± 6.43) |
|  |  |  |  |
| V4 (V1+21 days) | n | 6 | 8 |
|  | Twitch airway pressure (cmH2O) | 11 [4; 15]  9.83 (± 7.00) | 13 [8.5; 17.5]  12.50 (± 6.14) |
|  |  |  |  |
| V5 (V1+60 days) | n | 3 | 3 |
|  | Twitch airway pressure (cmH2O) | 14 [7; 16]  12.33 (± 4.73) | 10 [8; 13]  10.33 (± 2.52) |

Data are expressed as median [IQR], and mean (±SD)

Data are missing between V1 and V4 because patients were too sick to be moved

Supplemental Table 4: Forced vital capacity (% of theoretical value)

| Visit | Variable | Placebo N=17 | Amino Acid N=18 |
| --- | --- | --- | --- |
| V1 (Baseline) | N | 10 | 7 |
|  | FVC (%) | 27.5 [23; 35]  30.70 (± 11.15) | 47 [35; 58]  47.00 (± 13.01) |
|  |  |  |  |
| V2 (V1+7 days) | N | 10 | 13 |
|  | FVC (%) | 41.5 [34; 66]  49.10 (± 24.49) | 47 [39; 62]  49.92 (± 18.86) |
|  |  |  |  |
| V3 (V1+14 days) | N | 12 | 13 |
|  | FVC (%) | 52.5 [33; 75.5]  54.33 (± 29.35) | 60 [54; 71]  60.62 (± 17.32) |
|  |  |  |  |
| V4 (V1+21 days) | N | 11 | 13 |
|  | FVC (%) | 56 [44; 74]  59.09 (± 29.14) | 60 [53; 80]  67.23 (± 27.35) |
|  |  |  |  |
| V5 (V1+60 days) | N | 4 | 7 |
|  | FVC (%) | 60 [53.5; 87.5]  70.50 (± 27.53) | 68 [44; 78]  63.14 (± 16.53) |

Data are expressed as the median [IQR], and mean (±SD)

FVC: Forced vital capacity

Data are missing between V1 and V4 because patients were unable to participate

Supplemental Table 5: Urine 3 methyl-histidine (μmol /kg/24h)

| Visit | Variable | Placebo N=17 | Amino Acid N=18 |
| --- | --- | --- | --- |
| V1 (Baseline) | N | 17 | 16 |
|  | Urine methyl histidine (μmol /kg/24h) | 3.80 [2.28; 5.51]  3.91 (± 2.14) | 2.63 [2.00; 3.64]  2.96 (± 1.73) |
|  |  |  |  |
| V2 (V1+7 days) | N | 15 | 16 |
|  | Urine methyl histidine (μmol /kg/24h) | 3.03 [1.59; 5.21]  3.33 (± 2.39) | 2.03 [1.38; 4.32]  3.53 (± 4.87) |
|  |  |  |  |
| V3 (V1+14 days) | N | 11 | 12 |
|  | Urine methyl histidine (μmol /kg/24h) | 1.76 [1.11; 3.90]  2.53 (± 1.73) | 1.81 [1.13; 2.49]  2.07 (± 1.19) |
|  |  |  |  |
| V4 (V1+21 days) | n | 9 | 13 |
|  | Urine methyl histidine (μmol /kg/24h) | 2.33 [1.27; 2.70]  1.90 (± 0.96) | 2.25 [1.12; 2.62]  2.11 (± 1.10) |
|  |  |  |  |
| V5 (V1+60 days) | n | 3 | 3 |
|  | Urine methyl histidine (μmol /kg/24h) | 2.83 [1.08; 4.22]  2.71 (± 1.57) | 1.89 [1.72; 2.56]  2.06 (± 0.44) |

Data are expressed as the median [IQR], and mean (±SD)

Supplemental Table 6: Plasma I-FABP (pg/ml)

| Visit | Variable | Placebo N=17 | Amino Acid N=18 |
| --- | --- | --- | --- |
| V1 (Baseline) | N | 17 | 18 |
|  | Plasma I-FABP (pg/ml) | 650 [415; 1341]  983.1 (± 786.5) | 677.5 [487; 972]  6534.1 (± 24738.8) |
|  |  |  |  |
| V2 (V1+7 days) | N | 16 | 16 |
|  | Plasma I-FABP (pg/ml) | 1036.5 [753.5; 1461.5]  2401.0 (± 5122.7) | 873.5 [639.5; 1253.5]  2990.4 (± 8411.7) |
|  |  |  |  |
| V3 (V1+14 days) | N | 15 | 14 |
|  | Plasma I-FABP (pg/ml) | 889 [710; 1109]  975.0 (± 519.6) | 972.5 [763; 1475]  2031.8 (± 3809.0) |
|  |  |  |  |
| V4 (V1+21 days) | N | 15 | 14 |
|  | Plasma I-FABP (pg/ml) | 1137 [721; 1762]  1207.8 (± 613.5) | 902 [734; 1260]  1202.9 (± 1062.4) |
|  |  |  |  |
| V5 (V1+60 days) | N | 5 | 5 |
|  | Plasma I-FABP (pg/ml) | 540 [484; 1508]  874.8 (± 593.6) | 895 [834; 1476]  1124.2 (± 486.7) |

Data are expressed as the median [IQR], and mean (±SD)

Supplemental Table 7: Plasma citrulline (nmol/mL)

| Visit | Variable | Placebo N=17 | Amino Acid N=18 |
| --- | --- | --- | --- |
| V1 (Baseline) | N | 17 | 18 |
|  | Plasma citrulline (nmol/mL) | 17.95 [13.49; 21.38]  20.91 (± 12.28) | 16.78 [12.30; 21.17]  17.87 (± 8.44) |
|  |  |  |  |
| V2 (V1+7 days) | N | 16 | 16 |
|  | Plasma citrulline (nmol/mL) | 19.07 [15.91; 20.66]  19.27 (± 4.82) | 19.69 [16.17; 27.63]  21.27 (± 6.57) |
|  |  |  |  |
| V3 (V1+14 days) | N | 15 | 14 |
|  | Plasma citrulline (nmol/mL) | 18.21 [16.04; 21.13]  18.77 (± 4.71) | 22.57 [17.23; 28.27]  23.18 (± 7.68) |
|  |  |  |  |
| V4 (V1+21 days) | N | 15 | 14 |
|  | Plasma citrulline (nmol/mL) | 20.59 [16.62; 26.22]  20.97 (± 5.82) | 25.94 [19.42; 31.20]  26.59 (± 12.18) |
|  |  |  |  |
| V5 (V1+60 days) | N | 6 | 6 |
|  | Plasma citrulline (nmol/mL) | 17.51 [15.94; 18.23]  17.73 (± 5.30) | 24.62 [13.55; 35.08]  24.64 (± 10.94) |

Data are expressed as the median [IQR], and mean (±SD)

Supplemental Table 8: C-reactive protein (mg/L)

| Visit | Variable | Placebo N=17 | Amino Acid N=18 |
| --- | --- | --- | --- |
| V1 (Baseline) | N | 17 | 18 |
|  | C-reactive protein (mg/L)) | 124.1 [79 ;143.6]  116.12 (± 56.18) | 115.5 [61.6; 151.4]  106.45 (± 64.93) |
|  |  |  |  |
| V2 (V1+7 days) | N | 16 | 15 |
|  | C-reactive protein (mg/L) | 46.4 [27.9;140.6]  77.08 (± 73.85) | 28.2 [15.6 ; 60.3]  39.33 (± 34.19) |
|  |  |  |  |
| V3 (V1+14 days) | N | 15 | 14 |
|  | C-reactive protein (mg/L) | 35[7.1; 83.5]  55.83 (± 65.75) | 11.2 [4.5; 40.6]  28.07 (± 36.23) |
|  |  |  |  |
| V4 (V1+21 days) | N | 15 | 13 |
|  | C-reactive protein (mg/L) | 12.6 [4.5 ; 88.3]  42.27 (± 53.91) | 5.7 [2 ; 12.4]  21.09 (± 30.69) |
|  |  |  |  |
| V5 (V1+60 days) | N | 6 | 6 |
|  | C-reactive protein (mg/L) | 26.4 [2.5; 70.8]  35.22 (± 36.35) | 1.1 [0.7 ; 2.6]  13.44 (± 20.78) |

Data are expressed as the median [IQR], and mean (±SD)

Supplemental Table 9: Procalcitonin (µg/L)

| Visit | Variable | Placebo N=17 | Amino Acid N=18 |
| --- | --- | --- | --- |
| V1 (Baseline) | N | 17 | 18 |
|  | Procalcitonin (µg/L) | 0.39 [0.19 ; 1.92]  1.55 (± 2.33) | 0.39 [0.14 ; 1.59]  6.08 (± 13.06) |
|  |  |  |  |
| V2 (V1+7 days) | N | 16 | 16 |
|  | Procalcitonin (µg/L) | 0.12 [0.05 ; 0.31]  1.77 (± 4.70) | 0.1 [0.05 ; 0.31]  0.29 (± 0.40) |
|  |  |  |  |
| V3 (V1+14 days) | N | 15 | 14 |
|  | Procalcitonin (µg/L) | 0.05 [0.05 ; 0.23]  0.61 (± 1.36) | 0.06 [0.05 ; 0.14]  1.11 (± 3.78) |
|  |  |  |  |
| V4 (V1+21 days) | N | 15 | 14 |
|  | Procalcitonin (µg/L) | 0.05 [0.05 ; 0.15]  0.26 (± 0.46) | 0.05 [0.05 ;0.07]  0.09 (± 0.10) |
|  |  |  |  |
| V5 (V1+60 days) | N | 6 | 7 |
|  | Procalcitonin (µg/L) | 0.05 [0.05 ; 0.08]  0.22 (± 0.40) | 0.05 [0.05 ; 0.05]  0.05 (± 0.00) |

Data are expressed as the median [IQR], and mean (±SD)

Supplemental Table 10: Ferritin (ng/ml)

| Visit | Variable | Placebo N=17 | Amino Acid N=18 |
| --- | --- | --- | --- |
| V1 (Baseline) | N | 17 | 18 |
|  | Ferritin (ng/ml) | 341[132 ; 823.6]  476.3 (± 364.02) | 404.5 [168.4 ; 951.3]  11776 (± 46982.54) |
|  |  |  |  |
| V2 (V1+7 days) | N | 16 | 15 |
|  | Ferritin (ng/ml) | 507.1 [233.9 ; 768.1]  618.5 (± 520.45) | 296.9 [179.1 ;634.4]  430.1 (± 312.35) |
|  |  |  |  |
| V3 (V1+14 days) | N | 15 | 14 |
|  | Ferritin (ng/ml) | 324.1 [179.8 ; 542.9]  426.8 (± 351.13) | 365.3 [206.2; 531.2]  383.9 (± 230.27) |
|  |  |  |  |
| V4 (V1+21 days) | N | 15 | 13 |
|  | Ferritin (ng/ml) | 269.3 [137.9 ; 630.5]  396.3 (± 417.73) | 261.2 [229 ; 368.1]  296.5 (± 172.97) |
|  |  |  |  |
| V5 (V1+60 days) | N | 6 | 6 |
|  | Ferritin (ng/ml) | 513.9 [201.3 ;1007]  1274.5 (± 1999.6) | 127.8 [49.7 ;189.1]  127.2 (± 89.32) |

Data are expressed as the median [IQR], and mean (±SD)

Supplemental Table 11: Fibrinogen (g/L)

| Visit | Variable | Placebo N=17 | Amino Acid N=18 |
| --- | --- | --- | --- |
| V1 (Baseline) | N | 16 | 18 |
|  | Fibrinogen (g/L) | 6.00 [5.40 ; 7.55]  6.73 (± 2.11) | 5.75 [ 4.50; 7.30]  5.89 (± 1.99) |
|  |  |  |  |
| V2 (V1+7 days) | N | 16 | 15 |
|  | Fibrinogen (g/L) | 6.50 [5.65; 7.65]  6.73 (± 1.70) | 5.30 [4.20; 6.10]  5.51 (± 1.61) |
|  |  |  |  |
| V3 (V1+14 days) | N | 15 | 13 |
|  | Fibrinogen (g/L) | 5.60 [4.60; 7.40]  5.90 (± 1.47) | 4.60 [3.60; 5.20]  4.71 (± 1.56) |
|  |  |  |  |
| V4 (V1+21 days) | N | 15 | 15 |
|  | Fibrinogen (g/L) | 5.40 [4.00 ; 6.30]  5.08 (± 1.29) | 4.00 [2.90 ; 4.60]  4.21 (± 1.80) |
|  |  |  |  |
| V5 (V1+60 days) | N | 6 | 7 |
|  | Fibrinogen (g/L) | 5.25 [3.60 ; 6.00]  4.72 (± 1.85) | 2.70 [2.40 ; 2.80]  2.96 (± 0.92) |

Data are expressed as the median [IQR], and mean (±SD)

Supplemental Table 12: Feacal calprotectin (μg/g)

| Visit | Variable | Placebo N=17 | Amino Acid N=18 |
| --- | --- | --- | --- |
| V1 (Baseline) | N | 9 | 13 |
|  | Feacal calprotectin (μg/g) | 183.0 [71.0; 440.0]  236.0 (± 199.9) | 227.0 [117.0; 400.0]  303.2 (± 292.0) |
|  |  |  |  |
| V2 (V1+7 days) | N | 11 | 9 |
|  | Feacal calprotectin (μg/g) | 197.0 [74.0; 463.0]  253.1 (± 215.0) | 214.0 [126.0; 250.0]  298.6 (± 372.3) |
|  |  |  |  |
| V3 (V1+14 days) | N | 6 | 7 |
|  | Feacal calprotectin (μg/g) | 260.0 [85.0; 673.0]  341.3 (± 318.8) | 194.0 [38.0; 490.0]  263.4 (± 255.0) |
|  |  |  |  |
| V4 (V1+21 days) | N | 8 | 6 |
|  | Feacal calprotectin (μg/g) | 191.5 [123.5; 468.5]  299.3 (± 286.1) | 180.5 [39.0; 501.0]  259.0 (± 248.4) |
|  |  |  |  |
| V5 (V1+60 days) | N | 2 | 2 |
|  | Feacal calprotectin (μg/g) | 659 [18.0; 1300.0]  659.0 (± 906.5) | 107 [61.0; 153.0]  107.0 (± 65.1) |

Data are expressed as the median [IQR], and mean (±SD)

Supplemental Table 13: Alanine aminotransferase (UI/L)

| Visit | Variable | Placebo N=17 | Amino Acid N=18 |
| --- | --- | --- | --- |
| V1 (Baseline) | N | 17 | 18 |
|  | Alanine aminotransferase (UI/L) | 28 [22; 36]  37.8 (± 33.63) | 42.5 [20; 75]  504.4 (± 1877.64) |
|  |  |  |  |
| V2 (V1+7 days) | N | 16 | 15 |
|  | Alanine aminotransferase (UI/L) | 64.5 [37.5; 140]  96.4 (± 80.08) | 39 [21; 61]  47.5 (± 34.24) |
|  |  |  |  |
| V3 (V1+14 days) | N | 15 | 14 |
|  | Alanine aminotransferase (UI/L) | 52 [25; 79]  57.4 (± 41.98) | 31.5 [26; 49]  40.3 (± 31.49) |
|  |  |  |  |
| V4 (V1+21 days) | N | 15 | 13 |
|  | Alanine aminotransferase (UI/L) | 35 [21; 75]  49.1 (± 35.77) | 20 [15; 44]  39.8 (± 41.49) |
|  |  |  |  |
| V5 (V1+60 days) | N | 6 | 6 |
|  | Alanine aminotransferase (UI/L) | 20.5 [10; 35]  41.8 (± 58.29) | 30 [11; 50]  31.7 (± 24.44) |

Data are expressed as the median [IQR], and mean (±SD)

Supplemental Table 14: Alkaline phosphatase (UI/L)

| Visit | Variable | Placebo N=17 | Amino Acid N=18 |
| --- | --- | --- | --- |
| V1 (Baseline) | N | 17 | 18 |
|  | Alkaline phosphatase (UI/L) | 88 [60; 95]  93.8 (± 58.34) | 86 [65; 128]  111.2 (± 71.79) |
|  |  |  |  |
| V2 (V1+7 days) | N | 16 | 15 |
|  | Alkaline phosphatase (UI/L) | 109 [78.5; 156]  143.7 (± 94.19) | 89 [71 ; 116]  111.3 (± 74.96) |
|  |  |  |  |
| V3 (V1+14 days) | N | 15 | 14 |
|  | Alkaline phosphatase (UI/L) | 101 [87 ; 305]  158.7 (± 105.64) | 89 [62 ; 112]  103.8 (± 67.43) |
|  |  |  |  |
| V4 (V1+21 days) | N | 15 | 13 |
|  | Alkaline phosphatase (UI/L) | 102 [72; 218]  141.3 (± 85.22) | 81 [75; 104]  94.8 (± 43.48) |
|  |  |  |  |
| V5 (V1+60 days) | N | 6 | 6 |
|  | Alkaline phosphatase (UI/L) | 109 [63; 141]  117.3 (± 63.11) | 62.5 [51; 78]  60.2 (± 22.12) |

Data are expressed as the median [IQR], and mean (±SD)

Supplemental Table 15: Bilirubin (µmol/L)

| Visit | Variable | Placebo N=17 | Amino Acid N=18 |
| --- | --- | --- | --- |
| V1 (Baseline) | N | 17 | 18 |
|  | Bilirubin (µmol/L) | 6.6 [4; 8.1]  7.9 (± 7.04) | 6.6 [4.7; 10.1]  8.1 (± 5.03) |
|  |  |  |  |
| V2 (V1+7 days) | N | 16 | 15 |
|  | Bilirubin (µmol/L) | 7.9 [5.5; 10.1]  9.5 (± 7.78) | 6.2 [5.1; 7.4]  7.2 (± 4.59) |
|  |  |  |  |
| V3 (V1+14 days) | N | 15 | 14 |
|  | Bilirubin (µmol/L) | 7.3 [4.4; 9.6]  10.8 (± 15.04) | 5 [4; 9.4]  6.4 (± 3.02) |
|  |  |  |  |
| V4 (V1+21 days) | N | 15 | 13 |
|  | Bilirubin (µmol/L) | 5.7 [4.5; 7.8]  8.3 (± 9.7) | 4.8 [3.4; 8.2]  5.7 (± 2.99) |
|  |  |  |  |
| V5 (V1+60 days) | N | 6 | 6 |
|  | Bilirubin (µmol/L) | 7.2 [4.7; 10.4]  7.9 (± 3.89) | 7.1 [4.6; 12.2]  8.4 (± 4.51) |

Data are expressed as the median [IQR], and mean (±SD)

Supplemental Table 16: Pre-albumin (g/L)

| Visit | Variable | Placebo N=17 | Amino Acid N=18 |
| --- | --- | --- | --- |
| V1 (Baseline) | N | 17 | 18 |
|  | Pre-albumin (g/L) | 0.1 [0.1 ; 0.2]  0.1 (± 0.05) | 0.1 [0.1 ; 0.2]  0.1 (± 0.07) |
|  |  |  |  |
| V2 (V1+7 days) | N | 16 | 15 |
|  | Pre-albumin (g/L) | 0.2 [0.1 ; 0.3]  0.2 (± 0.07) | 0.2 [0.2 ; 0.3]  0.2 (± 0.09) |
|  |  |  |  |
| V3 (V1+14 days) | N | 15 | 14 |
|  | Pre-albumin (g/L) | 0.2 [0.1; 0.3]  0.2 (± 0.09) | 0.2 [0.2; 0.3]  0.2 (± 0.09) |
|  |  |  |  |
| V4 (V1+21 days) | N | 15 | 13 |
|  | Pre-albumin (g/L) | 0.2 [0.1; 0.3]  0.2 (± 0.09) | 0.3 [0.2; 0.3]  0.3 (± 0.08) |
|  |  |  |  |
| V5 (V1+60 days) | N | 6 | 6 |
|  | Pre-albumin (g/L) | 0.2 [0.1; 0.3]  0.2 (± 0.08) | 0.3 [0.3; 0.4]  0.4 (± 0.12) |

Data are expressed as the median [IQR], and mean (±SD)

Supplemental Table 17: Albumin (g/L)

| Visit | Variable | Placebo N=17 | Amino Acid N=18 |
| --- | --- | --- | --- |
| V1 (Baseline) | N | 17 | 18 |
|  | Albumin (g/L) | 24 [21; 26]  23.4 (± 3.64) | 22.5 [19; 26]  22.8 (± 5.96) |
|  |  |  |  |
| V2 (V1+7 days) | N | 16 | 15 |
|  | Albumin (g/L) | 24 [20; 28]  24.2 (± 6.8) | 26 [20; 32]  25.7 (± 6.75) |
|  |  |  |  |
| V3 (V1+14 days) | N | 15 | 14 |
|  | Albumin (g/L) | 26 [22; 33]  26.7 (± 7.94) | 29.5 [23; 32]  28.4 (± 6.68) |
|  |  |  |  |
| V4 (V1+21 days) | N | 15 | 13 |
|  | Albumin (g/L) | 32 [24; 35]  29.7 (± 8.12) | 32 [25; 34]  30.3 (± 7.2) |
|  |  |  |  |
| V5 (V1+60 days) | N | 6 | 6 |
|  | Albumin (g/L) | 27.5 [19; 37]  28.8 (± 9.89) | 37 [34; 41]  36.8 (± 3.97) |

Data are expressed as the median [IQR], and mean (±SD)

Supplemental Table 18: Threonine (nmol/ml)

| Visit | Variable | Placebo N=17 | Amino Acid N=18 |
| --- | --- | --- | --- |
| V1 (Baseline) | N | 17 | 18 |
|  | Threonine (nmol/ml) | 110.54 [86.43; 137.20]  133.89 (± 67.96) | 119.63 [90.22; 151.51]  130.98 (± 60.57) |
|  |  |  |  |
| V2 (V1+7 days) | N | 16 | 16 |
|  | Threonine (nmol/ml) | 107.04 [83.14; 159.67]  114.34 (± 42.49) | 222.63 [124.80; 354.33]  237.54 (± 125.52) |
|  |  |  |  |
| V3 (V1+14 days) | N | 15 | 14 |
|  | Threonine (nmol/ml) | 100.13 [78.34; 143.02]  111.67 (± 46.07) | 142.51 [128.77; 267.06]  207.55 (± 137.21) |
|  |  |  |  |
| V4 (V1+21 days) | N | 15 | 14 |
|  | Threonine (nmol/ml) | 97.44 [91.87; 114.04]  114.35 (± 41.43) | 124.94 [104.79; 158.06]  136.42 (± 52.25) |
|  |  |  |  |
| V5 (V1+60 days) | N | 6 | 6 |
|  | Threonine (nmol/ml) | 122.07 [104.60; 157.25]  126.51 (± 37.89) | 152.38 [120.83; 173.78]  144.16 (± 36.47) |

Data are expressed as the median [IQR], and mean (±SD)

Supplemental Table 19: Proline (nmol/ml)

| Visit | Variable | Placebo N=17 | Amino Acid N=18 |
| --- | --- | --- | --- |
| V1 (Baseline) | N | 17 | 18 |
|  | Proline (nmol/ml) | 172.10 [130.18; 261.26]  208.36 (± 117.38) | 202.05 [152.73; 222.75]  206.65 (± 102.36) |
|  |  |  |  |
| V2 (V1+7 days) | N | 16 | 16 |
|  | Proline (nmol/ml) | 153.38 [138.75; 200.32]  174.78 (± 69.55) | 208.74 [185.40; 324.79]  255.89 (± 122.46) |
|  |  |  |  |
| V3 (V1+14 days) | N | 15 | 14 |
|  | Proline (nmol/ml) | 145.47 [124.11; 232.39]  171.79 (± 70.72) | 239.06 [133.15; 325.70]  242.12 (± 105.67) |
|  |  |  |  |
| V4 (V1+21 days) | N | 15 | 14 |
|  | Proline (nmol/ml) | 178.74 [126.48; 205.29]  180.94 (± 67.11) | 209.66 [160.99; 256.67]  216.31 (± 63.97) |
|  |  |  |  |
| V5 (V1+60 days) | N | 6 | 6 |
|  | Proline (nmol/ml) | 238.87 [189.72; 266.66]  236.29 (± 43.78) | 284.22 [193.07; 309.51]  260.83 (± 106.49) |

Data are expressed as the median [IQR], and mean (±SD)

Supplemental Table 20: Serine (nmol/ml)

| Visit | Variable | Placebo N=17 | Amino Acid N=18 |
| --- | --- | --- | --- |
| V1 (Baseline) | N | 17 | 18 |
|  | Serine (nmol/ml) | 70.42 [59.67; 87.75]  78.22 (± 23.28) | 78.88 [67.83; 87.86]  77.13 (± 24.31) |
|  |  |  |  |
| V2 (V1+7 days) | N | 16 | 16 |
|  | Serine (nmol/ml) | 71.52 [54.39; 91.51]  73.80 (± 29.82) | 101.72 [87.07; 131.50]  111.84 (± 36.07) |
|  |  |  |  |
| V3 (V1+14 days) | N | 15 | 14 |
|  | Serine (nmol/ml) | 87.83 [54.47; 94.87]  81.95 (± 41.19) | 109.89 [85.17; 129.31]  113.43 (± 39.28) |
|  |  |  |  |
| V4 (V1+21 days) | N | 15 | 14 |
|  | Serine (nmol/ml) | 76.80 [59.95; 94.55]  78.17 (± 27.92) | 86.26 [75.51; 105.62]  88.43 (± 23.20) |
|  |  |  |  |
| V5 (V1+60 days) | N | 6 | 6 |
|  | Serine (nmol/ml) | 84.16 [62.28; 92.85]  87.37 (± 28.22) | 105.90 [100.16; 116.58]  106.68 (± 9.95) |

Data are expressed as the median [IQR], and mean (±SD)

Supplemental Table 21: Cysteine (nmol/ml)

| Visit | Variable | Placebo N=17 | Amino Acid N=18 |
| --- | --- | --- | --- |
| V1 (Baseline) | N | 17 | 18 |
|  | Cysteine (nmol/ml) | 48.53 [35.78; 54.84]  46.52 (± 14.37) | 42.09 [30.04; 47.27]  43.60 (± 18.60) |
|  |  |  |  |
| V2 (V1+7 days) | N | 16 | 16 |
|  | Cysteine (nmol/ml) | 55.66 [47.33; 63.58]  54.11 (± 16.42) | 64.40 [48.55; 74.93]  62.76 (± 18.61) |
|  |  |  |  |
| V3 (V1+14 days) | N | 15 | 14 |
|  | Cysteine (nmol/ml) | 54.18 [41.23; 55.94]  49.90 (± 10.50) | 68.82 [42.52; 78.58]  70.33 (± 31.38) |
|  |  |  |  |
| V4 (V1+21 days) | N | 15 | 14 |
|  | Cysteine (nmol/ml) | 46.78 [34.43; 55.17]  46.10 (± 11.48) | 58.26 [43.27; 65.13]  59.08 (± 24.91) |
|  |  |  |  |
| V5 (V1+60 days) | N | 6 | 6 |
|  | Cysteine (nmol/ml) | 59.19 [54.98; 64.04]  60.80 (± 9.75) | 58.16 [33.06; 67.87]  57.26 (± 28.04) |

Data are expressed as the median [IQR], and mean (±SD)

Supplemental Table 22: Leucine (nmol/ml)

| Visit | Variable | Placebo N=17 | Amino Acid N=18 |
| --- | --- | --- | --- |
| V1 (Baseline) | N | 17 | 18 |
|  | Leucine (nmol/ml) | 134.33 [109.90; 154.07]  133.12 (± 34.88) | 106.74 [86.74; 166.86]  129.26 (± 63.77) |
|  |  |  |  |
| V2 (V1+7 days) | N | 16 | 16 |
|  | Leucine (nmol/ml) | 108.28 [87.43; 142.09]  114.72 (± 37.65) | 147.90 [110.67; 175.75]  144.61 (± 37.83) |
|  |  |  |  |
| V3 (V1+14 days) | N | 15 | 14 |
|  | Leucine (nmol/ml) | 92.93 [65.78; 130.52]  107.34 (± 47.01) | 119.19 [95.53; 133.80]  118.33 (± 33.83) |
|  |  |  |  |
| V4 (V1+21 days) | N | 15 | 14 |
|  | Leucine (nmol/ml) | 92.50[78.83; 109.40]  96.73 (± 30.42) | 94.61 [83.45; 120.25]  98.89 (± 29.31) |
|  |  |  |  |
| V5 (V1+60 days) | N | 6 | 6 |
|  | Leucine (nmol/ml) | 101.98 [76.14; 129.57]  112.94 (± 45.36) | 116.83 [93.09; 138.89]  119.87 (± 27.02) |

Data are expressed as the median [IQR], and mean (±SD)

Supplemental Table 23: Arginine (nmol/ml)

| Visit | Variable | Placebo N=17 | Amino Acid N=18 |
| --- | --- | --- | --- |
| V1 (Baseline) | N | 17 | 18 |
|  | Arginine (nmol/ml) | 61.83 [44.40; 84.03]  67.31 (± 33.75) | 55.91 [46.72; 66.65]  54.95 (± 23.56) |
|  |  |  |  |
| V2 (V1+7 days) | N | 16 | 16 |
|  | Arginine (nmol/ml) | 72.02 [34.01; 82.01]  62.76 (± 25.74) | 64.73 [51.43; 77.15]  65.21 (± 15.77) |
|  |  |  |  |
| V3 (V1+14 days) | N | 15 | 14 |
|  | Arginine (nmol/ml) | 54.83 [33.32; 78.42]  57.98 (± 24.67) | 67.85 [59.02; 73.26]  69.23 (± 15.98) |
|  |  |  |  |
| V4 (V1+21 days) | N | 15 | 14 |
|  | Arginine (nmol/ml) | 59.35 [44.66; 74.97]  59.56 (± 20.34) | 62.13 [50.91; 70.31]  63.83 (± 17.14) |
|  |  |  |  |
| V5 (V1+60 days) | N | 6 | 6 |
|  | Arginine (nmol/ml) | 64.21 [47.48; 83.09]  67.92 (± 24.22) | 82.88 [56.84; 93.82]  78.31 (± 21.38) |

Data are expressed as the median [IQR], and mean (±SD)

Supplemental Table 24: Glutamine (nmol/ml)

| Visit | Variable | Placebo N=17 | Amino Acid N=18 |
| --- | --- | --- | --- |
| V1 (Baseline) | N | 17 | 18 |
|  | Glutamine (nmol/ml) | 435.19 [400.14; 520.98]  458.10 (± 112.09) | 472.43 [395.94; 582.84]  492.34 (± 148.23) |
|  |  |  |  |
| V2 (V1+7 days) | N | 16 | 16 |
|  | Glutamine (nmol/ml) | 427.52 [358.76; 475.30]  437.17 (± 127.58) | 497.23 [447.09; 545.49]  504.17 (± 78.92) |
|  |  |  |  |
| V3 (V1+14 days) | N | 15 | 14 |
|  | Glutamine (nmol/ml) | 470.18 [328.65; 537.47]  447.75 (± 102.54) | 525.90 [446.97; 603.70]  526.69 (± 91.43) |
|  |  |  |  |
| V4 (V1+21 days) | N | 15 | 14 |
|  | Glutamine (nmol/ml) | 499.34 [414.05; 536.78]  479.17 (± 61.90) | 505.97 [450.48; 536.79]  518.83 (± 90.58) |
|  |  |  |  |
| V5 (V1+60 days) | N | 6 | 6 |
|  | Glutamine (nmol/ml) | 565.49 [489.11; 581.32]  547.25 (± 51.62) | 568.64 [489.06; 666.49]  554.83 (± 119.85) |

Data are expressed as the median [IQR], and mean (±SD)

Supplemental Table 25: Zinc (ng/ml)

| Visit | Variable | Placebo N=17 | Amino Acid N=18 |
| --- | --- | --- | --- |
| V1 (Baseline) | N | 16 | 14 |
|  | Zinc (ng/ml) | 430.4 [339.3; 532.8]  446.8 (± 137.17) | 425.6 [351.9; 589]  461.3 (± 143.85) |
|  |  |  |  |
| V2 (V1+7 days) | N | 15 | 14 |
|  | Zinc (ng/ml) | 630.6 [466.3; 708.7]  595.2 (± 132.61) | 679 [592.9; 847.4]  704.7 (± 183.5) |
|  |  |  |  |
| V3 (V1+14 days) | N | 14 | 14 |
|  | Zinc (ng/ml) | 713.1 [563.1; 973.4]  737.3 (± 214.12) | 752.7 [582.2; 829]  739.5 (± 177.91) |
|  |  |  |  |
| V4 (V1+21 days) | N | 15 | 12 |
|  | Zinc (ng/ml) | 802.8 [634.6; 887.7]  787.1 (± 175.09) | 728.8 [660.5; 815]  722.3 (± 153.29) |
|  |  |  |  |
| V5 (V1+60 days) | N | 6 | 6 |
|  | Zinc (ng/ml) | 595.7 [543.7 ; 706.6]  612.7 (± 101.36) | 714.8 [674.6; 778]  753.8 (± 130.4) |

Data are expressed as the median [IQR], and mean (±SD)

Supplemental Table 26: Cholesterol (mmol/L)

| Visit | Variable | Placebo N=17 | Amino Acid N=18 |
| --- | --- | --- | --- |
| V1 (Baseline) | N | 17 | 18 |
|  | Cholesterol (mmol/L) | 3.1 [2.7; 3.5]  3.3 (± 1.24) | 3.2 [2.8; 3.9]  3.4 (± 0.96) |
|  |  |  |  |
| V2 (V1+7 days) | N | 16 | 15 |
|  | Cholesterol (mmol/L) | 3.4 [2.4; 3.8]  3.2 (± 0.98) | 3.8 [3.4; 4.4]  4 (± 0.72) |
|  |  |  |  |
| V3 (V1+14 days) | N | 15 | 14 |
|  | Cholesterol (mmol/L) | 3.4 [2.4; 4.5]  3.5 (± 1.2) | 4.3 [3.3; 4.9]  4.3 (± 1.14) |
|  |  |  |  |
| V4 (V1+21 days) | N | 15 | 13 |
|  | Cholesterol (mmol/L) | 4.6 [2.5; 5.4]  4 (± 1.54) | 4.8 [3.8; 5.1]  4.6 (± 0.86) |
|  |  |  |  |
| V5 (V1+60 days) | N | 6 | 6 |
|  | Cholesterol (mmol/L) | 4.2 [2.8; 5.1]  4.1 (± 1.3) | 5.6 [5; 5.7]  5.3 (±1.35) |

Data are expressed as the median [IQR], and mean (±SD)
